# KRAS^G12D^ mutation promotes pancreatic tumorigenesis by suppressing sirtuin three *via* the guanine nucleotide exchange factor RCC1

**DOI:** 10.1016/j.jbc.2025.111057

**Published:** 2025-12-13

**Authors:** Taoyi Mai, Mengwen Wang, Ya Qiu, Wenhua Lu, Hongyu Wu, Shuna Chen, Paul J. Chiao, Peng Huang

**Affiliations:** 1 Sun Yat-Sen University Cancer Center, State Key Laboratory of Oncology in South China, Collaborative Innovation Center for Cancer Medicine, Guangzhou, China; 2 The Eighth Affiliated Hospital, Sun Yat-Sen University, Shenzhen, Guangdong, China; 3 Department of Pharmacology, Molecular Cancer Research Center, School of Medicine, Sun Yat-Sen University, Shenzhen, China; 4 Department of Cellular & Molecular Oncology, The University of Texas MD Anderson Cancer Center, Houston, Texas, USA

**Keywords:** gene regulation, KRAS, pancreatic cancer, RCC1, SIRT3

## Abstract

KRAS^G12D^ mutation is a prevalent gain-of-function mutation that drives pancreatic cancer tumorigenesis, but the underlying mechanisms that promote KRAS-induced cell proliferation and tumor formation remain elusive. To uncover the molecular pathways that facilitate KRAS^G12D^-driven malignant transformation, we measured the transcriptomic alterations at various time points after induction of KRAS^G12D^ expression in human pancreatic normal epithelial cells. KEGG pathway enrichment of the differentially expressed genes (DEGs) showed that the major DEGs were located in pathways that regulate nicotinate/nicotinamide metabolism, TNF signaling, and microRNAs associated with cancer. Among these molecular alterations, the NAD-dependent deacetylase gene *SIRT3* was significantly down-regulated by KRAS^G12D^. Conversely, forced overexpression of SIRT3 inhibited pancreatic cancer cell proliferation both *in vitro* and *in vivo*. Mechanistic study identified RCC1 as a key molecule that mediated KRAS^G12D^ inhibition of *SIRT3* transcription. Knockdown of RCC1 in pancreatic cancer cells restored SIRT3 expression and impaired tumor formation *in vivo*. Overall, our study has revealed a previously unrecognized mechanism by which oncogenic KRAS promotes tumor development through down-regulation of the SIRT3-mediated tumor suppression pathway, and has also identified RCC1 as a potential therapeutic target for treatment of cancer patients with KRAS mutations.

Pancreatic cancer is one of the most lethal malignant diseases with a 5-year survival rate of only approximately 13% (1). The lack of distinct symptoms and effective early diagnosis techniques results in most pancreatic cancer patients being diagnosed at advanced stages with metastasis or unsuitable for surgery due to critical vascular involvement (2). Pancreatic cancer cells possess strong survival capacity even under stress microenvironment, contributing to chemotherapy resistance and the very poor 5-year survival rate for the patients (3, 4). Thus, research on early pancreatic cancer pathogenesis to uncover the key molecules that promote pancreatic cancer development will provide new mechanistic insights that serve as a basis to develop more effective targeted therapeutic strategies to improve the survival rate of pancreatic cancer patients.

The gain-of-function *KRAS* mutation is the primary driver of pancreatic cancer development. Approximately 92% of pancreatic cancer tissues carry various types of activating *KRAS* mutants, which appear at the early stage of the disease development (5). Among these mutants, KRAS^G12D^ accounts for approximately 40% of all KRAS mutation in pancreatic cancer, making it the most prevalent subtype (6). Compared to other subtypes, KRAS^G12D^ mutation in pancreatic cancer is associated with a higher degree of malignant cellular behaviors (7). Although the relationship between KRAS^G12D^ and the onset/initiation of pancreatic cancer has been clarified through studies on genetically engineered mouse models, the specific carcinogenic mechanisms, especially other molecular alterations that help promoting KRAS-driven cancer progression, remains unclear (8).

Sirtuin 3 is a nicotinamide adenine dinucleotide (NAD)-dependent deacetylase belonging to the sirtuin family (9). SIRT3 is mainly located in the mitochondria and participates in the regulation of metabolic pathways such as the tricarboxylic acid cycle, oxidative phosphorylation, fatty acid oxidation, and oxidative stress response by regulating the acetylation levels of certain metabolic enzymes (10). The roles of SIRT3 are diverse among different cancer types and genetic subtypes. SIRT3 was shown to impair tumor growth by inhibiting glycolysis and cell proliferation or by promoting apoptosis in breast, lung, and colon cancer (11, 12, 13, 14). In contrast, it SIRT3 was suggested to promote the development of acute myeloid leukemia, diffuse large B cell lymphoma, breast, lung, and colon cancer with various genetic backgrounds (15, 16, 17, 18, 19). In pancreatic cancer, patients with low SIRT3 expression seem to exhibit a rapid recurrence and short survival, while high SIRT3 expression could inhibit *in vitro* cell proliferation (20, 21, 22). The exact role of SIRT3 in pancreatic cancer tumorigenesis *in vivo* and the relation between KRAS and SIRT3 remain to be investigated.

In this study, we used the human pancreatic normal epithelial (HPNE) cells harboring an inducible KRAS^G12D^ expression system to investigate the transcriptomic alterations after induction of KRAS^G12D^ for various time periods, aiming to identify molecular changes that facilitate KRAS^G12D^-driven cancer growth. We found that SIRT3 was significantly down-regulated by KRAS activation. Overexpression of SIRT3 inhibited pancreatic cancer proliferation *in vitro* and tumorigenesis *in vivo*. We further showed that the regulator of chromosome condensation 1 (RCC1) mediated the KRAS^G12D^ inhibition of SIRT3 transcription. Knockdown of RCC1 in PANC-1 cells promoted the expression of SIRT3 and impaired tumorigenesis capacity. These new findings indicate that the down regulation of the RCC1/SIRT3 axis plays a significant role in KRAS-driven pancreatic cancer development.

## Results

### Activation of KRAS^G12D^ leads to transcriptomic changes associated with altered nicotinate/nicotinamide metabolism

**Figure 1:**
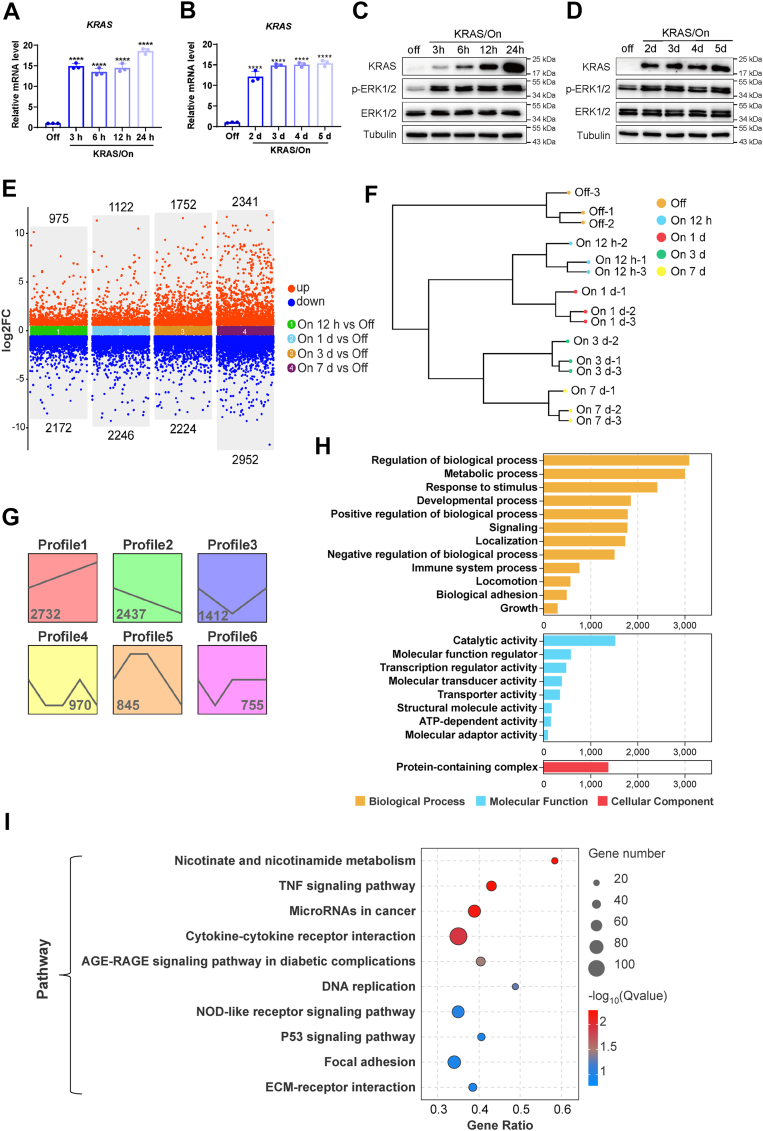
**Transcriptomic analysis in HPNE cells before and after induction of KRAS^G12D^ expression.***A, KRAS* mRNA levels of HPNE KRAS/Off, HPNE KRAS/On 3 h, 6 h, 12 h, and 24 h cells. The data are presented as mean ± SD (*n* = 3). *p* values were determined by Student’s *t* test. ∗∗∗∗, *p* < 0.0001. *B, KRAS* mRNA levels of HPNE KRAS/Off, HPNE KRAS/On 2 days, 3 days, 4 days, and 5 days cells. The data are presented as mean ± SD (*n* = 3). *p* values were determined by Student’s *t* test. ∗∗∗∗, *p* < 0.0001. *C,* KRAS and ERK1/2 protein levels as well as the ERK1/2 phosphorylation level of HPNE KRAS/Off, HPNE KRAS/On 3 h, 6 h, 12 h, and 24 h cells. Cell extracts were analyzed by Western blotting using tubulin as the loading control. *D,* KRAS and ERK1/2 protein levels as well as the ERK1/2 phosphorylation levels of HPNE KRAS/Off, HPNE KRAS/On 2 days, 3 days, 4 days, and 5 days cells. Cell extracts were analyzed by Western blotting using tubulin as the loading control. *E,* scatter plot of differentially expressed genes (DEGs) of HPNE KRAS/On 12 h, 1 day, 3 days, and 7 days cells *versus* HPNE KRAS/Off cells, respectively. *F,* cluster dendrogram of the transcriptome. *G,* significantly enriched expression patterns of trend analysis of DEGs from (*E*). The polyline within each trend block represents the gene expression pattern; the number in the *lower right* corner represents the number of genes included. *H,* GO enrichment of genes from “profile 1” (*red*) and “profile 2” (*green*) from (*G*). *I,* KEGG pathway enrichment of genes from “profile 1” (*red*) and “profile 2” (*green*) from (*G*). HPNE: human pancreatic normal epithelial cell; DEG: differentially expressed gene.

To investigate the molecular pathways involved in KRAS^G12D^-driven malignant transformation of pancreatic ductal cells, we first analyzed gene expression changes in HPNE cells before and after KRAS^G12D^ activation, using an inducible KRAS^G12D^ cell system we previously described (23). The expression of KRAS mRNA and protein in HPNE cells was significantly induced as early as 3 h after exposure to doxycycline, and the KRAS^G12D^ expression remained highly elevated for many days as long as doxycycline was present (Fig. 1, *A*–*D*). This elevated KRAS^G12D^ expression led to an activation of the downstream MEK/ERK signaling pathway, evidenced by a substantial increase in phosphorylation of ERK1/2 without any increase in ERK1/2 total protein (Fig. 1, *C* and *D*), indicating that the forced expression of KRAS^G12D^ was functional in the cells.

We then used RNA-Seq to perform transcriptomic analysis of gene expression profiles at multiple time points after KRAS^G12D^ induction. Compared to the KRAS/Off cells, the overall transcription patterns of HPNE were significantly altered in the KRAS^G12D^/On cells (Fig. 1*E*). Differentially expressed genes (DEGs) between KRAS/On and KRAS/Off HPNE cells increased in a time-dependent manner after KRAS^G12D^ induction (Fig. 1, *E* and *F*). Trend analysis of DEGs revealed rather complex patterns of genes expression changes, including genes whose expression gradually increased (profile 1), gradually decreased (profile 2), decreased and then increased (profile 3), and more complex profiles (Figs. 1*G*, S1*A*). Among the 20 profiles, profiles #1 and #2 contained genes that were respectively up-regulated or down-regulated by KRAS^G12D^. GO enrichment of “profile 1” and “profile 2” showed that DEGs were significantly enriched in the biological process regulation, metabolic process, stimulus response, catalytic enzymes, and molecular regulators (Fig. 1*H*). KEGG pathway analysis showed that nicotinamide and nicotinate metabolism is the most significant enriched pathway (Fig. 1*I*), which contained DEGs involved in the synthesis or regulation of NAD metabolism (Fig. S1*B*). In addition to this novel finding, the differentially expressed genes were also enriched in TNF signaling, cytokine interactions, microRNAs in cancer, p53 signaling pathways and other cellular processes (Fig. 1*I*), which were consistent with the previous reports on KRAS regulation of gene expression (24, 25, 26, 27).

### SIRT3 is transcriptionally down-regulated by KRAS^G12D^

**Figure 2:**
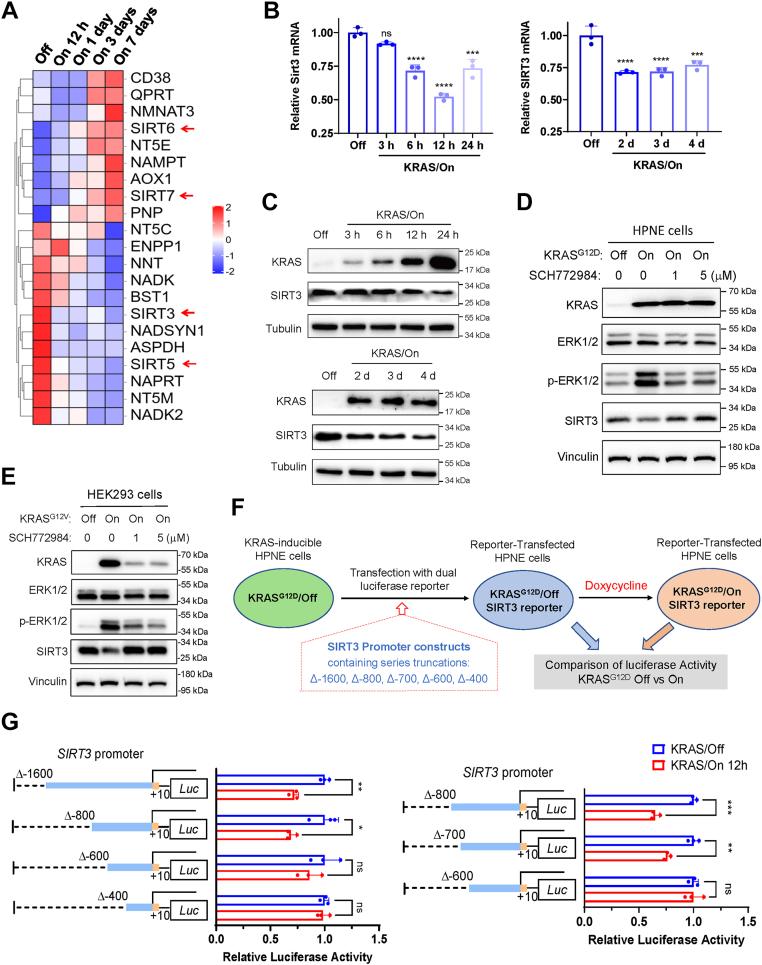
**SIRT3 is transcriptionally down-regulated by KRAS^G12D^.***A,* differential gene expression of nicotinate and nicotinamide metabolism pathways. *B, SIRT3* mRNA levels of HPNE cells with KRAS/Off or KRAS/On for 3 h to 96 h. The data are presented as mean ± SD (*n* = 3). *p* values were determined by Student’s *t* test. ∗∗∗, *p* < 0.001; ∗∗∗∗, *p* < 0.0001. *C,* SIRT3 and KRAS protein levels in HPNE cells with KRAS/Off or KRAS/On for 3 h to 96 h. Cell extracts were analyzed by Western blotting using tubulin as the loading control. Note that the images of KRAS and tubulin bands were derived from the same source images shown in Figure 1, *C* and *D*, as they were from the same experiment. *D,* effect of SCH772984 on expression of ERK1/2, phosphorylated ERK1/2, and SIRT3 protein levels in KRAS/On HPNE cells. Cells were treated with SCH772984 at indicated concentrations for 24 h. Cell extracts were analyzed by Western blotting using vinculin as the loading control. HPNE KRAS/Off cell extracts were used for comparison. *E,* effect of SCH772984 on expression of ERK1/2, phosphorylated ERK1/2, and SIRT3 protein levels in KRAS/On HEK293 cells. Cells were treated with SCH772984 at indicated concentrations for 24 h. Cell extracts were analyzed by Western blotting using vinculin as the loading control. HEK293 KRAS/Off cell extracts were used for comparison. *F,* schematic illustration of the promoter activity assay to identify potential KRAS regulated region in the SIRT3 promoter. *G,* activity of truncated SIRT3 promoters in HPNE KRAS/Off and HPNE KRAS/On (12 h) cells. The data are presented as mean ± SD (*n* = 3). *p* values were determined by Student’s *t* test. ∗, *p* < 0.05;∗∗, *p* < 0.01. HPNE: human pancreatic normal epithelial cell.

Since analysis of the KRAS-induced transcriptomic data revealed that the DEGs were highly enriched in the nicotinamide and nicotinate metabolism (Fig. 1*I*), we further used heat map analysis to evaluate the RNA-seq data, and identified four NAD-dependent molecules belonging to the Sirtuin family (SIRT3, SIRT5, SIRT6, SIRT7) that were differentially expressed in the KRAS^G12D^-induced (KRAS^G12D^/On) cells, with *SIRT3* and *SIRT5* down-regulated while *SIRT6* and *SIRT7* up-regulated after induction of KRAS^G12D^ expression (Figs. 2*A*, S2). Further quantitative reverse transcription polymerase chain reaction analysis showed that *SIRT3* was significantly down-regulated as early as 6 h after KRAS activation (Fig. 2*B*). Western blotting also revealed a time-dependent decrease in SIRT3 protein (Fig. 2*C*). Interestingly, the increase in SIRT6 and SIRT7 mRNA expression occurred with some delay (24–72 h after KRAS activation, Fig. S2), suggesting that that such increase might likely be a cellular response to the early decrease of SIRT3 (6 h) and SIRT5 (12 h) induced by KRAS activation. Since knockout of SIRT5 has been reported to promote the KRAS^G12D^-driven PDAC progression in the KPC mouse model (28), we mainly focused our study on the regulation of SIRT3 by KRAS^G12D^ and its potential role in KRAS-driven pancreatic cancer development.

To explore the possible downstream pathway that mediated KRAS down-regulation of SIRT3 expression, we used a chemical inhibitor SCH772984 to suppress the ERK phosphorylation, a main signaling pathway downstream of KRAS, and then tested its effect on SIRT3 expression. SCH772984 (1–5 μM) significantly inhibited KRAS-induced ERK phosphorylation without affecting the total ERK protein as expected (Fig. 2*D*). Importantly, the originally suppressed expression of SIRT3 protein in the KRAS/On cells was almost completely restored after SCH772984 treatment, as evidenced by an increase of SIRT3 protein in the SCH772984-treated KRAS^G12D^/On cells to the level comparable to that of the KRAS^G12D^/Off cells (Fig. 2*D*), indicating that KRAS suppressed SIRT3 expression *via* the ERK signaling pathway. Similar results were also observed in HKE293 cells containing an inducible KRAS^G12V^ (Fig. 2*E*), suggesting that this regulatory mechanism was not limited to G12D mutation, and could be observed in other KRAS mutations.

To identify the potential transcriptional factor(s) mediating the KRAS^G12D^-induced down-regulation of SIRT3, we first constructed a dual luciferase reporter system containing the *SIRT3* promoter DNA sequence with a series of progressive truncations from −1800 bp to −400 bp to locate the DNA domain responsive to KRAS^G12D^ activation. As shown in Figure 2*F*, HPNE cells harboring the inducible KRAS^G12D^ in “Off” stage (without doxycycline) were first transfected with the dual luciferase reporter constructs containing *SIRT3* promoter DNA with the indicated truncations, and a portion of the transfected cells was then exposed to doxycycline to induce KRAS expression. Luciferase activity in the KRAS/Off and KRAS/On cells was measured and compared to map the DNA domain in the SIRT3 promoter responsible for the difference in transcription activity between the two KRAS stages (On or Off). The initial analysis using the first batch of constructs with progressive truncations mapped the DNA domain spanning between −600 bp to −800 bp (Fig. 2*G*, left panel). Further analysis using constructs with 100-bp truncations identified the KRAS responsive element located between −700 to −600 bp region (Fig. 2*G*, right panel).

We then performed *in silico* motif analysis of the *SIRT3* promoter by using JASPAR to seek potential transcription factors that might mediate the *SIRT3* inhibition. Potential cis-regulatory elements that were predicted to be recognized by KRAS-activated transcription factors include v-myc avian myelocytomatosis viral oncogene homolog (MYC), AP-1, and ELK1 (Fig. S3*A*). Of note, these three motifs were also found in the *SIRT5* promoter. The MYC binding site was located in the −699 to −688 bp region of the *SIRT3* promoter, and an AP-1 binding site was found in the −689 to −683 bp region. To test the potential role of MYC and AP-1 in regulation of SIRT3 expression, we treated HPNE/On cells with MYC inhibitor MYCi361 and AP-1 inhibitor T-5224. As shown in Figure S3*B*, MYCi361 significantly reduced MYC protein level but did not alter SIRT3 expression in KRAS/On cells. Similarly, T-5224 did not alter the expression of SIRT3 in KRAS-On cells (Fig. S3*C*), suggesting that neither MYC nor AP-1 had any significant role in KRAS suppression of SIRT3 expression. This prompted us to further search for the molecule(s) responsible for KRAS^G12D^-induced downregulation of SIRT3.

### Down-regulation of SIRT3 by KRAS^G12D^ is mediated *via* RCC1

**Figure 3:**
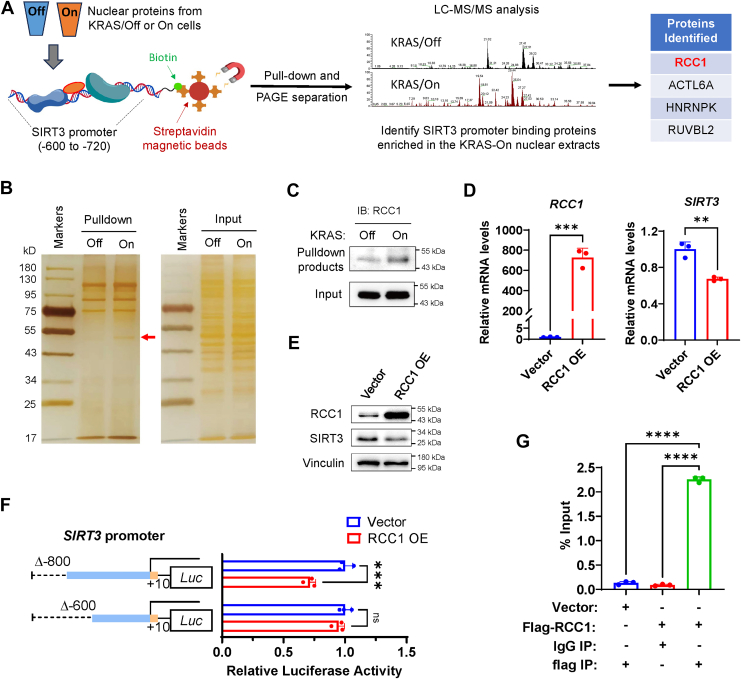
**Down-regulation of SIRT3 by KRAS^G12D^ is mediated *via* RCC1.***A,* schematic illustration of the study design to identify potential KRAS regulated *SIRT3* promoter binding proteins. *B,* silver staining of the proteins binding to the *SIRT3* promoter DNA. The biotin-labeled DNA probe spanning −720 to −600 bp of the SIRT3 promoter and the nuclear protein extracts from the KRAS/Off or KRAS/On (12 h) cells were used in the DNA pull-down assay. The pulldown products were run on an SDS-PAGE. After electrophoresis, the proteins in the gel were revealed by silver staining. *C,* RCC1 protein levels pulled-down by the *SIRT3* promoter DNA probe from the nuclear protein extracts of the KRAS/Off or KRAS/On (12 h) cells. *D, RCC1* and *SIRT3* mRNA levels in RCC1 overexpressed and control HPNE cells. The data are presented as mean ± SD (*n* = 3). *p* values were determined by Student’s *t* test. ∗∗, *p* < 0.01; ∗∗∗, *p* < 0.001. *E,* RCC1 and SIRT3 protein levels in RCC1 overexpressed and control HPNE cells. *F,* activity of the −800 bp and −600 bp SIRT3 promoter in the RCC1 overexpressed and control HPNE cells. The data are presented as mean ± SD (*n* = 3). *p* values were determined by Student’s *t* test. ∗∗∗, *p* < 0.001. *G,* enrichment level of −700 to −600 bp *SIRT3* promoter in the RCC1 chromatin co-immunoprecipitation. Flag-tagged RCC1 was transfected to pull-down the bond DNA, followed by qPCR using the primers spanning −700 to −600 bp of the SIRT3 promoter for the determination of the enrichment level. The data are presented as mean ± SD (*n* = 3). *p* values were determined by Student’s *t* test. ∗∗∗∗, *p* < 0.0001. HPNE: human pancreatic normal epithelial cell; RCC1, regulator of chromosome condensation 1.

To further identify the protein factor that binds to the KRAS^G12D^ responsive DNA domain in *SIRT3* promoter, we constructed a biotin-conjugated DNA with sequence identical to the *SIRT3* promoter region between −600 and −720, and used this DNA probe to pull down its binding proteins from the nuclear extracts of KRAS/Off or KRAS/On cells (Fig. 3*A*). Electrophoresis (SDS-PAGE) of the pulled down proteins showed a band of protein with molecular weight of approximately 45 to 50 kDa that was substantially enriched in the KRAS/On cells (Fig. 3*B*). Mass spectrometry analysis of the proteins in this band region identified four molecules including RCC1, ACTL6A, HNRNPK, and RUVBL2 (Fig. 3*A*).

To further test which of these candidates regulates SIRT3, we first used Western blotting to measure the level of HNRNPK pulled-down by *SIRT3* promoter DNA, and found that the HNRNPK band was located at approximately 65 kDa (Fig. S4*A*), which was much larger than the differentially enriched band (45–50 kDa) in the KRAS/On cells (Fig. 3*B*). Also, since the HNRNPK protein levels were similar in the KRAS/On and KRAS/Off cells, this molecule unlikely mediated KRAS regulation of SIRT3. To evaluate the impact of the remaining three molecules (RCC1, ACTL6A, RUVBL2) on *SIRT3* expression, they were separately over-expressed in KRAS/Off cells. Analysis of SIRT3 mRNA showed that overexpression of RCC1, ACTL6A, or RUVBL2 could all inhibit SIRT3 expression (Fig. S4*B*). Analysis of The Cancer Genome Atlas (TCGA) datasets revealed that high expression of RCC1 or ACTL6A was significantly correlated with poor survival of pancreatic cancer patients (*p* < 0.05), whereas RUVBL2 expression was not correlated with the patient survival (*p* = 0.4524, Fig. S4*C*). These data suggest that RUVBL2 was unlikely play a significant role in promoting the malignant phenotype induced by KRAS. We then compared the effect of RCC1 and ACTL6A on SIRT3 protein expression, and found that a knockdown of RCC1 substantially elevated SIRT3 protein (Fig. S4*D*), whereas knockdown of ACTL6A only caused a modest increase in SIRT3 protein (Fig. S4*E*), suggesting that RCC1 likely played a key role in mediating KRAS regulation of SIRT3 expression.

RCC1 was enriched in the KRAS/On cells by *SIRT3* promoter pull-down followed by Western blot analysis (Fig. 3*C*). We then tested if RCC1 could functionally regulate the expression of SIRT3, and found that overexpression of RCC1 in HPNE cells led to a significant inhibition of SIRT3 expression at mRNA level (Fig. 3*D*) and protein level (Fig. 3*E*). Consistently, RCC1 knockdown in HPNE cells significantly induced SIRT3 expression (Fig. S4*D*), suggesting that RCC1 could indeed mediate the suppression of SIRT3 by KRAS^G12D^. To further validate the ability of RCC1 to suppress SIRT3 expression, we used the *SIRT3* promoter reporter system to test the effect of RCC1 overexpression on the reporter activity (luciferase), and found that overexpression of RCC1 significantly inhibited the activity of SIRT3 promoter between −800 bp and −600 bp region (Fig. 3*F*). Chromatin immunoprecipitation (ChIP) assay further demonstrated the intrinsic ability of RCC1 to bind the *SIRT3* promoter DNA at −700 bp to −600 bp region (Fig. 3*G*). These data together indicate that activation of KRAS^G12D^ led to inhibition of *SIRT3* transcription by recruiting RCC1 to the *SIRT3* promoter at −700 bp to −600 bp region.

### Suppression of SIRT3 expression promote pancreatic cancer growth

**Figure 4:**
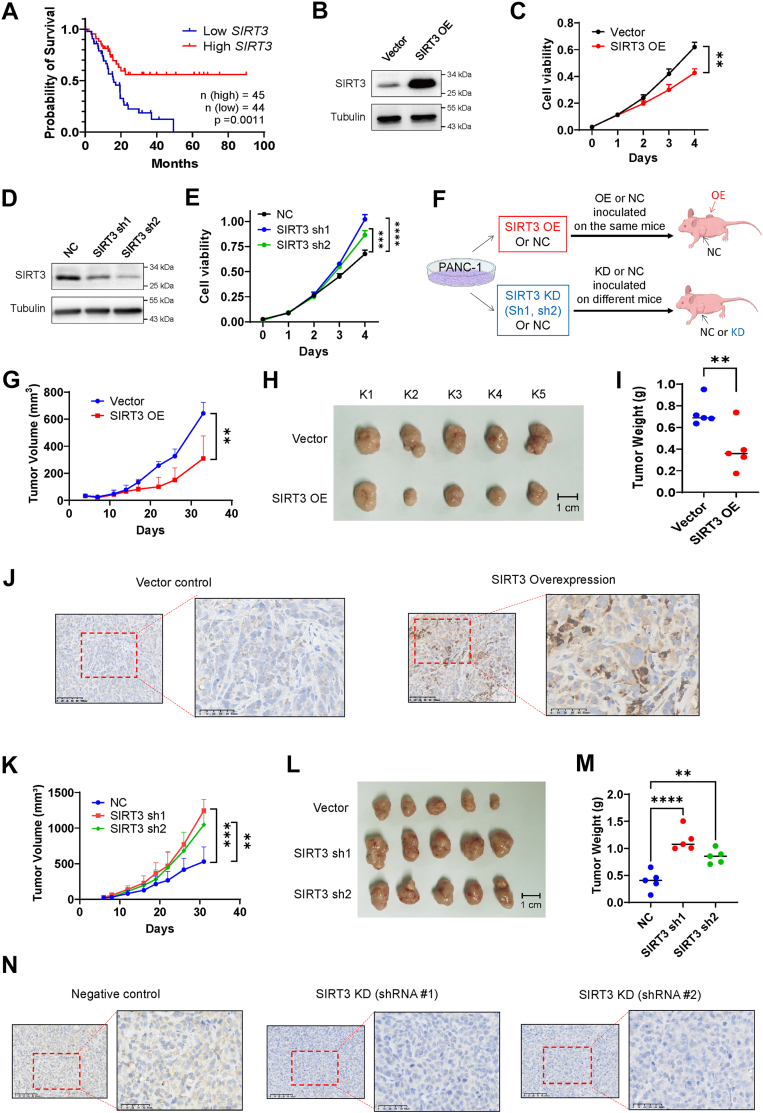
**SIRT3 suppresses pancreatic cancer growth *in vitro* and *in vivo*.***A,* kaplan-meier survival plot of pancreatic cancer patients with high and low *SIRT3* expression at 75%/25% cutoff from The Cancer Genome Atlas database. *B,* SIRT3 protein levels in SIRT3 overexpressed (SIRT3 OE) and control (Vector) PANC-1 cells. *C,* growth curves of PANC-1 Vector cells and PANC-1 SIRT3 OE cells *in vitro*. The data are presented as mean ± SD (*n* = 3). *p* values were determined by Student’s *t* test. ∗∗, *p* < 0.01. *D,* SIRT3 protein levels in SIRT3 knockdown (SIRT3 sh1, sh2) and control (NC) PANC-1 cells. *E,* growth curves of PANC-1 NC, SIRT3 sh1 and sh2 cells *in vitro*. The data are presented as mean ± SD (*n* = 3). *p* values were determined by Student’s *t* test. ∗∗∗, *p* < 0.001; ∗∗∗∗, *p* < 0.0001. *F,* schematic illustration of study design to evaluate the impact of SIRT3 on PANC1 tumor growth *in vivo*. *G,* growth curve of SIRT3 overexpressed (SIRT3 OE) and control (Vector) PANC1 subcutaneous tumors. The data are presented as mean ± SD (*n* = 5). *p* values were determined by Student’s *t* test. ∗∗, *p* < 0.01. *H*, PANC1 subcutaneous tumors of the SIRT3 overexpression and control group. The characters at the top of the photo represent the mouse number. The length of the scale bar represents 1 cm. *I,* SIRT3 overexpressed and the control PANC1 tumor weight. The *red* and blue points connected by the line represent tumors inoculated on each side of the same mouse. *p* values were determined by Student’s *t* test. ∗∗, *p* < 0.01. *J,* SIRT3 immunohistochemistry of the SIRT3 overexpressed and the control PANC1 subcutaneous tumors. The images are representative sections of tumor #2 shown in Figure S6*A*, which contains the IHC images of all tumors including the same source images shown here. The length of the scale bar represents 100 μm. *K,* Growth curve of SIRT3 knockdown (SIRT3 sh1, sh2) and control (NC) PANC1 subcutaneous tumors. The data are presented as mean ± SD (*n* = 5). *p* values were determined by Student’s *t* test. ∗∗, *p* < 0.01; ∗∗∗, *p* < 0.001. *L,* PANC1 subcutaneous tumors of the SIRT3 knockdown and control group. *M,* SIRT3 knockdown and the control PANC1 tumor weight. The data are presented as mean ± SD (*n* = 5). *p* values were determined by Student’s *t* test. ∗∗, *p* < 0.01; ∗∗∗∗, *p* < 0.0001. *N,* SIRT3 immunohistochemistry of the SIRT3 knockdown and the control PANC1 subcutaneous tumors. The images are representative sections of tumors #2 and #3 in Figure S6*B*, which contains the IHC images of all tumors including the same source images shown here. The length of the scale bar represents 100 μm.

Since conflicting/opposite impacts of SIRT3 on tumor growth have been reported in the literature (11, 15, 29, 30), we thus performed patient survival analysis as well as *in vitro* and *in vivo* experiments to evaluate the role of SIRT3 on pancreatic cancer development in the context of KRAS activation. Analysis of pancreatic cancer patient survival data in the TCGA database revealed that patients with high *SIRT3* expression exhibited better overall survival compared to those with lower low SIRT3 expression (Fig. 4*A*), suggesting that SIRT3 might have suppressive effect on pancreatic cancer. Indeed, transfection experiments showed that overexpression of SIRT3 in pancreatic cancer cells (PANC1) significantly inhibited cell proliferation (Fig. 4, *B* and *C*), whereas knockdown of SIRT3 by shRNA enhanced cell growth *in vitro* (Fig. 4, *D* and *E*). Consistently, overexpression of SIRT3 in the KRAS/On HPNE cells also significantly retarded cell growth (Fig. S4, *A* and *B*). Similar results were also observed in pancreatic cancer cell line (AsPC-1) with KRAS^G12D^ mutation (Fig. S4, *C* and *D*). Conversely, a knockdown of SIRT3 in AsPC-1 promoted cell proliferation (Fig. S4, *E* and *F*)

We then performed *in vivo* tumor formation assay by pairwise injecting SIRT3-overexpressed PANC1 cells and the vector-transfected PANC1 cells as the control (NC) to each side of the same mice, or by inoculating PANC1 cells with shRNA-mediated stable knockdown of SIRT3 in comparison with control PANC1 cells (Fig. 3*F*). The results showed that SIRT3 overexpression significantly reduced the tumor growth (Fig. 4, *G*–*I*). Immunohistochemistry staining of the tumor samples confirmed that SIRT3 protein levels were substantially increased in the SIRT3-OE xenografts (Figs. 4*J*, S6*A*). In contrast, a stable knockdown of SIRT3 by shRNA significantly promoted tumor growth *in vivo* (Fig. 4, *K*–*M*). Immunohistochemistry staining of these mouse tumor samples confirmed that SIRT3 protein levels were indeed decreased *in vivo* (Figs. 4*N*, S6*B*). Together, both *in vitro* and *in vivo* results demonstrated that SIRT3 functioned as a tumor suppressor in KRAS^G12D^-driven pancreatic cancer.

Interestingly, we found that the levels of SIRT3 expression significantly affected the sensitivity of pancreatic cancer cells to MRTX1133, a KRAS^G12D^ inhibitor in phase I/II clinical trial (31). Overexpression of SIRT3 significantly enhanced the cytotoxicity of MRTX1133 against pancreatic cancer cells (PANC1) in a colony formation assay (Fig. S7*A*), whereas SIRT3 knockdown by shRNA reduced the sensitivity of PANC1 cells to MRTX1133 treatment (Fig. S7*B*). For instance, MRTX1133 at the concentration of 1 μM inhibited 59% colony formation in the control PANC1 cells, whereas the same concentration of MRTX1133 inhibit 78% colony formation in PANC1 cells with SIRT3 overexpression (*p* = 0.047). In contract, a knockdown of SIRT3 resulted in decrease of the drug sensitivity (39–41% inhibition, *p* = 0.039). Thus, it appeared that the down-regulation of SIRT3 expression by KRAS^G12D^ might be a mechanism by which pancreatic cancer cells become resistance to the KRAS^G12D^ inhibitor.

### Knockdown of RCC1 impaired the tumor growth of pancreatic cancer

**Figure 5:**
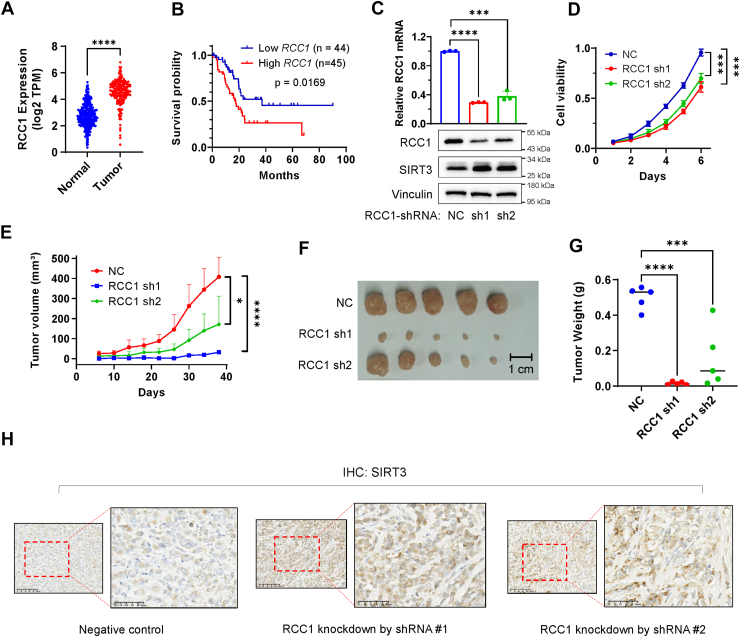
**Abrogation of RCC1 suppresses pancreatic cancer cell proliferation and tumor growth through up-regulation of SIRT3.***A,* RCC1 gene expression levels in normal pancreas and pancreatic cancer tissues. RCC1 expression in normal pancreas was acquired from the GTEx database, and the expression in pancreatic cancer tissue was acquired from The Cancer Genome Atlas database. *p* values were determined by Welch's *t* test. ∗, *p* < 0.05. *B,* kaplan-meier survival plot of pancreatic cancer patients with high and low *RCC1* expression at a 75%/25% cutoff from The Cancer Genome Atlas database. *C, RCC1* mRNA level and RCC1 and SIRT3 protein levels of PANC1 NC, RCC1 sh1 and sh2 cells. The data are presented as mean ± SD (*n* = 3). *p* values were determined by Student’s *t* test. ∗∗∗, *p* < 0.001; ∗∗∗∗, *p* < 0.0001. *D,* growth curves of PANC1 NC, RCC1 sh1, and sh2 cells *in vitro*. The data are presented as mean ± SD (*n* = 3). *p* values were determined by Student’s *t* test. ∗∗∗, *p* < 0.001. *E,* growth curve of RCC1 knockdown (RCC1 sh1, RCC1 sh2) and control (NC) PANC1 subcutaneous tumors. The data are presented as mean ± SD (*n* = 5). *p* values were determined by Student’s *t* test. ∗, *p* < 0.05; ∗∗∗∗, *p* < 0.0001. *F,* PANC1 subcutaneous tumors of RCC1 knockdown group and control group. The length of the scale bar represents 1 cm. *G,* RCC1 knockdown and the control PANC1 tumor weight. The data are presented as mean ± SD (*n* = 5). *p* values were determined by Student’s *t* test. ∗∗∗, *p* < 0.001; ∗∗∗∗, *p* < 0.0001. *H,* SIRT3 immunohistochemistry of RCC1 knockdown and control PANC1 subcutaneous tumors. The images are representative sections of tumor #1 in Figure S8*C*, which contains the full IHC images of all tumors including the same source images shown here. The length of the scale bar represents 100 μm. RCC1, regulator of chromosome condensation 1.

Considering our new finding that KRAS down-regulated SIRT3 through the guanine exchange factor RCC1 *in vitro* (Fig. 3), we thus further evaluated the role of RCC1 in affecting pancreatic cancer cell proliferation and tumor growth *in vivo*. First, analysis of TCGA database showed that the *RCC1* expression was significantly higher in pancreatic cancer than in normal pancreas tissue (Fig. 5*A*). Importantly, high expression of *RCC1* was significantly correlated with poor clinical outcome, as evidenced by lower overall survival in pancreatic cancer patients with high RCC1 expression (Fig. 5*B*), suggesting that RCC1 might play a major role in pancreatic cancer development.

We then used shRNA to knockdown the expression of RCC1 in PANC1 cells, and tested its effect on cell proliferation in culture and tumor formation *in vivo*. As shown in Figure 5*C*, RCC1 shRNA#1 and shRNA#2 were both effective in down-regulating RCC1 expression, leading to an elevated expression of SIRT3 protein (Fig. 5*C*, lower panel), consistent with the suppressive effect of RCC1 on SIRT3 expression (Fig. 3). The knockdown of RCC1 expression caused a significant decrease in cell proliferation of PANC1 and AsPC-1 cells (Figs. 5*D*, S8, *A* and *B*). In animal experiments, stable knockdown of RCC1 expression in PANC1 cells potently suppressed their ability to grow tumors *in vivo* (Fig. 5, *E*–*G*). IHC staining of the tumor tissues showed that the RCC1 knockdown caused an increase in SIRT3 protein expression *in vivo* (Figs. 5*H*, S8*C*).

To further investigate whether the suppressive effect of RCC1 knockdown on tumor cell proliferation was mediated by SIRT3, we performed a SIRT3 knockdown in the RCC1-knockdown PANC1 cells. The proliferation of the RCC1-knockdown cells was significantly inhibited, and this inhibition was partially rescued by SIRT3 knockdown (Fig. S8*D*), indicating that SIRT3 contributed at least in part to the RCC1-induced inhibition of cell proliferation.

## Discussion

Patients with pancreatic cancer exhibits the highest mortality rate among all cancer types. The poor prognosis of pancreatic cancer can be attributed to multiple factors including lack of early diagnosis technology, intrinsic cellular resistance to chemotherapeutic drugs, and immunosuppressive tumor microenvironment (2, 4). We found that the overexpression of KRAS^G12D^ dramatically altered the transcriptome of HPNE cells within 12 h (Fig. 1, *E* and *F*). Interestingly, the number of down-regulated genes was twice the number of the up-regulated genes in the early time point (12 h). As the cells adapt to the high expression of KRAS^G12D^, the number of up-regulated genes eventually increased as time progresses (3-7 days). These results indicated that the activating KRAS mutation in human pancreatic cells could dramatically regulate the expression of many genes, consistent with the observation in mouse pancreas cells (32). The differentially expressed genes in the KRAS/On cells exhibited multiple expression patterns with respect to time of induction (Figs. 1*G*, S1*A*), suggesting that complex mechanisms are involved in the gene expression regulation and maintenance of homeostasis.

GO enrichment of the DEGs revealed that changes in cell metabolism, stress, and development processes were closely related to the KRAS^G12D^-driven cellular transformation involving regulation of transcription, translation, and catalytic functions of metabolic enzymes and signaling factors (Fig. 1*H*). In addition, the KEGG analysis significantly enriched multiple pathways that have been reported to be closely related to pancreatic cancer development such as the TNF signaling pathway and AGE-RAGE signaling pathway (Fig. 1*I*) (24, 25, 33). Studies on genetically engineered mouse models found that the formation of pancreatic cancer involved a combination of pancreatitis and KRAS gain-of-function mutation (34, 35). The elevated expression of pro-inflammatory factors IL-6 and IL-33 observed in our KRAS-on cells suggest that KRAS^G12D^ might also be a driving factor of pancreas inflammatory (35, 36).

Among the enriched pathways, nicotinate and nicotinamide metabolism exhibited the most significant changes in the KEGG analysis (Fig. 1*G*). The differentially expressed genes in this metabolic pathway mainly involve in the synthesis and catabolism of NAD, a redox cofactor participating in the regulation of cellular metabolism, protein post-translational modification, and signal transduction. Several enzymes in NAD metabolic pathway such as nicotinamide phosphoribosyl transferase (NAMPT), NAD + kinase, and the NAD-dependent sirtuin family have been reported to be altered in pancreatic cancer. Of note, NAMPT1, the rate-limiting enzyme of the NAD salvage pathway, was up-regulated by KRAS^G12D^ activation in our study. Consistently, other studies showed that inhibition of NAMPT1 expression attenuated pancreatic cancer growth *in vivo* (37), and that NAD + kinase could be phosphoryl-activated by KRAS/PKC axis to support pancreatic cancer growth (38).

A novel finding from this study was the discovery that KRAS^G12D^ induces major changes in expression of the sirtuin deacetylase family members, especially the down-regulation of SIRT3. Previous studies suggest that the impact of sirtuin family members on cancer development and drug resistance might vary among different SIRT members, and likely depend on cell types. For instance, SIRT1 and SIRT7 were reported to promote pancreatic cancer progression (39, 40), while SIRT2, SIRT4, SIRT5, and SIRT6 seem to play a tumor suppressor role (28, 41, 42, 43). The effect of SIRT3 on pancreatic cancer development largely remains unclear. Correlative studies showed that pancreatic cancer patients with low SIRT3 expression exhibited higher degree of malignancy with poor clinical prognosis (20, 44). However, the relationship between SIRT3 and KRAS and their mechanistic link remain unknown. Our study showed that overexpression of SIRT3 could significantly inhibit the proliferation of pancreatic cancer cells *in vitro* and suppress tumor growth *in vivo*, whereas suppression of SIRT3 expression by shRNA significantly promoted pancreatic cell proliferation *in vitro* and tumor growth in mice. These data demonstrate the tumor-suppressive effect of SIRT3 against pancreatic cancer. Importantly, we found that induction of KRAS^G12D^ expression led to a significant decrease in SIRT3 expression (Fig. 2), suggesting that down-regulation of SIRT3 by KRAS might be a mechanism that facilitates KRAS-driven pancreatic cancer development.

Another significant finding of this study was the elucidation of the mechanism by which KRAS down regulated SIRT3 expression. KRAS gain-of-function mutations, including G12D, G12C and G12V are known to activate RAF/MEK/ERK pathway in various cancer types (45). We showed that inhibition of ERK phosphorylation rescued the repression of SIRT3 expression in KRAS/On HPNE cells, suggesting that the ERK signaling pathway mediated the down-regulation of SIRT3 by KRAS^G12D^. In a HEK293 cell line with inducible KRAS^G12V^ (46), the induction of KRAS^G12V^ also inhibited SIRT3 expression, and ERK phosphorylation inhibitor SCH772984 treatment restored the expression of SIRT3 (Fig. S3*D*), suggesting ERK signaling might be a common pathway by which KRAS gain-of-function mutations suppresses SIRT3 suppression.

Through DNA pull-down of the SIRT3 promoter-bond protein followed by electrophoresis separation and mass spectrometry analysis, we identified RCC1 as a regulatory molecule that mediated KRAS down-regulation of SIRT3, based on its ability to bind SIRT3 promoter (−600 to −800 bp), its iBAQ value, its molecular weight, and validation by Western blot analysis using specific antibody. As such, our study revealed a novel regulatory axis linking KRAS and SIRT3 through RCC1, although the exact mechanism by which RCC1 inhibit SIRT3 still remains unclear.

RCC1 is a guanine-nucleotide exchange factor that was initially identified as a regulator for the onset of chromosome condensation at the level of mRNA transcription or maturation (47). Elevated expression of RCC1 has been observed in cervical cancer, colon cancer, and soft tissue sarcoma, where RCC1 may play important role in nucleo-cytoplasmic trafficking of certain molecules such as Skp2 to regulate cell cycle transition and proliferation (48, 49, 50). However, role RCC1 in pancreatic cancer remained clear. Our study showed that RCC1 could mediate the down-regulation of SIRT3 expression in KRAS-driven cancer cells. RCC1 is known to bind to chromosome by interacting with the nucleosome core particle as a dynamic complex, where It may contact nucleosomal DNA by its DNA-binding loop and N-terminal tail (51). Of note, the ability of RCC1 to regulate the expression of a specific gene such as *SIRT3* has not been reported previously. In our study, DNA pull-down and ChIP assay demonstrated a direct interaction of RCC1 and *SIRT3* promoter, leading to suppression of SIRT3 expression. However, the possibility of RCC1 to function as a direct transcriptional repressor is still speculative, and the exact mechanism by which RCC1 repressed SIRT3 transcription remain elusive. This limitation of mechanistic insights calls for further investigation in this area.

A knockdown of RCC1 in PANC-1 cells could significantly upregulate SIRT3 expression and inhibited cancer cell proliferation *in vitro* and tumor growth *in vivo* (Fig. 5, *C*–*I*), indicating a critical role of RCC1 in pancreatic cancer development. As such, RCC1 could be a potential target for the treatment of pancreatic cancer. However, due to the inability of HPNE-KRAS cells to form tumor in mice, we were unable to use HPNE cells to test the functional roles of RCC1 and SIRT3 *in vivo*. The use of only PANC-1 tumor model in animal experiments is a limitation of this study. Also, due to a lack of potential binding pocket in the RCC1 protein, developing RCC1-specific inhibitors or/and PROTAC-base degraders could be challenging. To our knowledge, currently there is no specific RCC1 inhibitor for potential cancer treatment. Thus, development of RCC1-targeted drugs remains as a major task for future study.

The successful development of drugs specifically targeting KRAS represents a major progress in targeted therapy in recent years. These mutant-specific KRAS-targeted drugs, including Sotorasib, Adagrasib, and MRTX1133, provide new hope for patients suffering from highly malignant tumors driven by *KRAS* mutations such as pancreatic cancer, lung cancer, and colon cancer. However, intrinsic and acquired resistance to KRAS-targeted drugs has been reported, and presents a major challenge (31). Mechanisms contributing to such drug resistance includes the upregulation of RTK-RAS pathway genes and EMT regulators, and oncogenic NFE2L2^D29H^ mutation (52). In our study, we found that a knockdown of SIRT3 by shRNA in pancreatic cancer cells led to a significant resistant to MRTX1133, whereas overexpression of SIRT3 enhanced the sensitivity of pancreatic cancer cells to MRTX1133 (Fig. S7). These results demonstrate that SIRT3 may play an important role in affecting the sensitivity of pancreatic cancer cells to the KRAS^G12D^ inhibitor, and suggest that the down-regulation of SIRT3 by KRAS^G12D^ may be an intrinsic mechanism by which pancreatic cancer cells harboring KRAS^G12D^ resists its own inhibitor. As such, up-regulation of SIRT3 by abrogating RCC1 might by a potentially effective strategy to overcome resistance to this drug in pancreatic cancer. However, the mechanism by which SIRT3 modulates cellular sensitivity to MRTX1133 still remains unclear. This lack of mechanistic insights presents a limitation to the development of effective therapeutic strategies to overcome cancer resistance to KRAS-targeted drugs, and calls for further study in this important area.

## Experimental procedures

### Cell culture, chemicals, and reagents

hTERT-HPNE, HEK293, HEK293T PANC-1 and AsPC-1 cells were purchased from ATCC. HPNE and HEK293 cells were cultured in Dulbecco’s Modified Eagle’s Medium (DMEM) with 10% (v/v) tetracycline-free fetal bovine serum (C2720, VivaCell). HEK293T and PANC-1 was cultured in DMEM with 10% (v/v) fetal bovine serum (C04001, VivaCell). AsPC-1 was cultured in RPMI-1640 with 10% (v/v) fetal bovine serum. Both cells were maintained at 37 °C in a 5% CO^2^ environment. The culture conditions for the inducible KRAS^G12D^ HPNE cells and inducible KRAS^G12V^ HEK293 cells were described previously (23, 46). For the induction of KRAS^G12D^ or KRAS^G12v^ expression, the respective cells were treated with 100 ng/ml doxycycline (HY-N0565A, MedChemExpress). The ERK phosphorylation inhibitor SCH772984 (HY-50846), MYC inhibitor MCYi361 (HY-129600) and AP-1 inhibitor T-5224 (HY-12270) were purchased from MedChemExpress.

### Plasmid construction and cell transfection

The shRNA of SIRT3 and RCC1 were constructed into pLKO.1 vector backbone using Quick Ligation Kit (M2200L, New England Biolabs). Coding sequence of SIRT3 was constructed into pLVX vector backbone, and coding sequences of RCC1, RUVBL2 and ACTL6A were constructed into pcDNA 3.1 vector backbone, using ClonExpress II One Step Cloning Kit (C112–02, Vazyme). SIRT3 promoters were constructed into pGL3 vector backbone using ClonExpress II One Step Cloning Kit. The RCC1 siRNA were purchased from RuiBiotech Biotechnology Co. Ltd. The shRNA and siRNA sequences are listed in Table S1.

The shRNA and SIRT3 OE plasmid were respectively transfected with PAX and PMD2G plasmid into HEK293T cells using jetPRIME (101000001, Polyplus-transfection) according to the manufacturer’s protocol, to generate lentivirus. After 48 h incubation, culture media with the virus were collected and added to HPNE/Off or PANC-1 cells for 24 h transfection. SIRT3 promoter, RCC1 OE, RUVBL2 OE and ACTL6A OE plasmids were transfected into HPNE cells using Lipofectamine 3000 (L3000075, Thermo Fisher Scientific) according to the manufacturer’s protocol. The RCC1 siRNA were transfected in HPNE cells using Lipofectamine RNAiMAX (13778075, Thermo Fisher Scientific) according to the manufacturer’s protocol. Cells were harvested after transfection for further experiments.

### RNA extraction and quantitative reverse transcription polymerase chain reaction (qRT-PCR)

Cellular RNA was extracted using Trizol (15596018CN, Thermo Fisher Scientific) followed by the manufacturer’s protocol. Purified RNA was reverse-transcribed into cDNA using the Color Reverse Transcription Kit (A0010CGQ, EZBioscience). Color SYBR Green qPCR Mix (A0012, EZBioscience) was used to detect and quantify target mRNA levels. Quantitative PCR was performed as previously described (23). The primers used in the quantitative reverse transcription polymerase chain reaction are listed in Table S2.

### Western blot analysis

Western blot analysis was performed as previously described (23). Antibodies specific for the following markers were purchased from the indicated suppliers: SIRT3 (10099-1-AP, Proteintech; 1:1000), RCC1 (22142-1-AP, Proteintech; 1:1000), ERK (4695, Cell Signaling Technology; 1:1000), p-ERK (4370, Cell Signaling Technology; 1:1000), Vinculin (4650, Cell Signaling Technology; 1:1000), α-Tubulin (2144, Cell Signaling Technology; 1:1000), KRAS (sc-30, Santa Cruz; 1:1000). Blots were developed by Clarity Max Western ECL Substrate (1705062, Bio-Rad) and captured by ChemiDoc Imaging System (Bio-Rad).

### RNA-seq

The total RNA samples were sent to Gene Denovo Biotechnology Co. Ltd for RNA-seq analysis. The process of cDNA library construction was performed as described: The mRNA with polyA was enriched using Oligo (dT) beads and fragmented by ultrasonic. Using the mRNA fragments as templates and random oligonucleotides as primers to synthesize double-stranded cDNA, carrying out terminal repair, and adding A tail and ligation sequencing. cDNAs with a length of approximately 200 bp were screened for PCR amplification and purified by AMPure XP beads. The cDNA library was sequenced using Illumina Novaseq 6000. Processing and analysis of RNA-seq data were performed on the Omicsmart platform (www.omicsmart.com, Gene Denovo Biotechnology).

### Promoter activity assay

Promoter activity assay was performed using the Dual-Luciferase Reporter Assay System (E1910, Promega). In brief, cells transfected with promoter-pGL3 plasmid and pRL-TK plasmid were washed with PBS. Passive lysis buffer was added to each well and the cell lysates were collected. Luciferase activity was measured according to the manufacturer’s protocol by Synergy H1 microplate reader (Biotek). Firefly-luciferase activity of the promoter construct was normalized to the Renilla-luciferase activity.

### DNA pull-down assay

The biotinylated probe of the SIRT3 promoter (forward sequence: CATCCTGGCTACAGCTCTTGGCCCTCGTGGTCTGTCATCCCTCTTATTCCTCGACCCTGCCACTCAAGGTTCCTCAGCAAACCTTGCCGGCACAGCCGGTCGCTACACGTCCCCGTTTCC) was immobilized to Dynabeads MyOne Streptavidin T1 (65601, Thermo Fisher Scientific) according to the manufacturer’s procedure. Nuclear protein was extracted using NE-PER nuclear and cytoplasmic extraction reagents (78833, Thermo Fisher Scientific). Mix nuclear protein and Dynabeads in pull-down binding buffer (1.17 g NaCl, 40 μl NP-40, 4 ml 1M Tris-HCl, adjust pH to 7.4 and add ddH_2_O to 40 ml) and incubate overnight at 4 °C with stirring by a rotator. The protein-DNA-beads were washed with pull-down washing buffer (117 mg NaCl, 100 μl NP-40, 4 ml 1M Tris-HCl, 5 ml glycerol, adjust pH to 7.4 and add ddH_2_O to 200 ml) three times, then the DNA-binding proteins were eluted with RIPA lysis buffer.

### Protein mass spectrometry

Polyacrylamide gel was developed by Pierce Silver Stain for Mass Spectrometry (24600, Thermo Fisher Scientific) according to the manufacturer’s procedure. The appropriate size of bands was cut and sent to Shanghai Bioprofile Biotechnology Co., Ltd for mass spectrometry identification.

Samples were decolorized in a 1:1 (v/v) mixture of 300 mM K_3_Fe(CN)_6_ and 100 mM Na_2_S_2_O_3_. To each sample, proteins were reduced in 40 μl of 100 mM DTT and 360 μl of 100 mM NH_4_HCO_3_ at 56 °C for 30 min. In-gel digestion was performed by 20 ng/μl trypsin in 50 mM NH_4_HCO_3_ buffer overnight at 37 °C. Digested peptides were desalted and concentrated using a C18 StageTip column and dried under vacuum.

For LC-MS/MS analysis, peptides were reconstituted in 0.1% formic acid aqueous solution. Peptides from each sample were separated using a nanoflow Easy nLC 1200 chromatography system (Thermo Fisher Scientific). Mobile phases: Buffer A (0.1% formic acid in water) and Buffer B (0.1% formic acid in 80% acetonitrile/water). The column was equilibrated with 100% Buffer A. The sample was loaded onto a Trap Column (100 μm × 20 mm, 5 μm C18, Dr Maisch GmbH) and separated on an analytical column (75 μm × 150 mm, 3 μm C18, Dr Maisch GmbH) with a gradient elution at 300 nl/min. The gradient program was as follows: 0 to 2 min: 2% to 5% Buffer B; 2 to 44 min: 5% to 28% Buffer B; 44 to 51 min: 28% to 40% Buffer B; 51 to 53 min: 40% to 100% Buffer B; 53 to 60 min: 100% Buffer B.

Peptides were analyzed using a Q Exactive Plus mass spectrometer (Thermo Fisher Scientific) in data-dependent acquisition mode. Parameters were set as indicated: Duration: 60 min; Polarity: Positive ion mode; MS1 scan range: 350 to 1800 m/z; MS1 resolution: 60,000 @ m/z 200; AGC target: 3e6; Max injection time (MS1): 50 ms; MS2: Top 20 most intense ions per full scan; MS2 resolution: 15,000 @ m/z 200; AGC target (MS2): 1e5; Max injection time (MS2): 50 ms; HCD activation, isolation window: 1.6 m/z; Normalized collision energy: 28%.

The mass spectrometry data were analyzed using MaxQuant 2.0.1.0 utilizing the protein database: UniProt Reference Proteome *Homo sapiens* (Human) [9606] 81,791_20230317.fasta (https://www.uniprot.org/proteomes/UP000005640). This database was downloaded on March 17, 2023, and contained 81,791 protein entries. The parameter settings for MaxQuant analysis were set as: Enzyme: Trypsin; Max Missed Cleavages: 2; Precursor Tolerance (Main search): 4.5 ppm; Precursor Tolerance (First search): 20 ppm; Fixed modifications: Carbamidomethyl (C); Variable modifications: Oxidation (M), Acetyl (Protein N term); Database pattern: Target Reverse; PSM FDR:0.01; Protein FDR: 0.01; Site FDR: 0.01. Identified peptides and proteins are listed in Table S3A and S3B.

### Chromatin co-immunoprecipitation (ChIP)

Chromatin co-immunoprecipitation was performed using Pierce Magnetic ChIP Kit (26157, Thermo Fisher Scientific). In brief, cells were fixed with formaldehyde for 15 min with a final concentration of 1%, and the fixation was terminated with glycine. The cells were washed with PBS and cell precipitation was suspended with IP Dilution Buffer. After centrifugation, the cells were lysed with MNase and then sonicated according to the manufacturer’s procedure. Appropriate IgG or DYKDDDDK Tag antibodies (14793, Cell Signaling Technology) were added into the suspension and incubate at 4 °C overnight. ChIP Grade Protein A/G Magnetic beads were then added for another 2 h incubation at 4 °C. Samples were washed with IP Wash Buffer and de-crosslinked with NaCl and proteinase K at 65 °C. DNA was purified by a purification column provided by the Pierce Magnetic ChIP Kit.

### Cell viability assay

Cell viability was measured using MTS. Cells were seeded in 96-well plates (1 × 10^3^ cells in 200 μl medium per well). After incubation at 37 °C in the indicated periods, MTS reagent (G1111, Promega) was added into each well (20 μl/well) and cells were incubated for 2 h at 37 °C. Results were subsequently read at 490 nm by the Synergy H1 microplate reader (Biotek).

### Tumor formation assay

All mouse experiment procedures were performed using protocols reviewed and approved by the Animal Care and Use Committee of Sun Yat-sen University Cancer Center. For the tumorigenesis assay of SIRT3 overexpressed PANC-1, 2.5 × 10^6^ vector control and SIRT3-OE PANC-1 cells were subcutaneously injected on the left and right side of the same female BALB/c nude mice, respectively. For the tumorigenesis assay of SIRT3 and RCC1 knockdown PANC-1, 2.5 × 10^6^ cells were subcutaneously injected on the left side of female BALB/c nude mice. 5 days after cell injection, the long and short diameters of tumors were recorded twice a week. When the tumor volume (long diameter × short diameter^2^ ÷ 2) of the control group reached about 500 mm^3^, the mice were euthanized and the tumors were dissected for weight measurement.

### Immunohistochemical staining

Mouse xenograft tumors were fixed in 4% paraformaldehyde and embedded in paraffin. Immunohistochemical staining was performed as previously described (23). Sections were used for H&E staining or immunostaining with primary anti-SIRT3 (10099-1-AP, Proteintech; dilution, 1:250) or anti-RCC1 (22142-1-AP, Proteintech; dilution, 1:800) antibodies.

## Data availability

All data are contained within the article and supporting material. Further information and requests for resources and reagents should be directed to the lead contact, Huang Peng (huangpeng@sysucc.org.cn).

## Supporting information

This article contains supporting information.

## Conflict of interest

The authors declare that they have no conflicts of interest with the contents of this article.
