# Supplementary material for: KRASG12D mutation promotes pancreatic tumorigenesis by suppressing sirtuin three via the guanine nucleotide exchange factor RCC1
Source: J Biol Chem. 2025 Dec 13;302(2):111057. doi: 10.1016/j.jbc.2025.111057 (PMC12804135; doi:10.1016/j.jbc.2025.111057)
Supplement: Supporting information [file mmc1.pdf]

KRAS^{G12D} mutation promotes pancreatic tumorigenesis by suppressing Sirtuin 3 via the guanine nucleotide exchange factor RCC1

Taoyi Mai, Mengwen Wang, Ya Qiu, Wenhua Lu, Hongyu Wu, Shuna Chen, Paul J Chiao, Peng Huang

List of Supporting Information:

1.Supplementary Figure S1
2.Supplementary Figure S2
3.Supplementary Figure S3
4.Supplementary Figure S4
5.Supplementary Figure S5
6.Supplementary Figure S6
7.Supplementary Figure S7
8.Supplementary Figure S8
9.Supplementary Table S1
10.Supplementary Table S2

Supplementary Figure S1

A

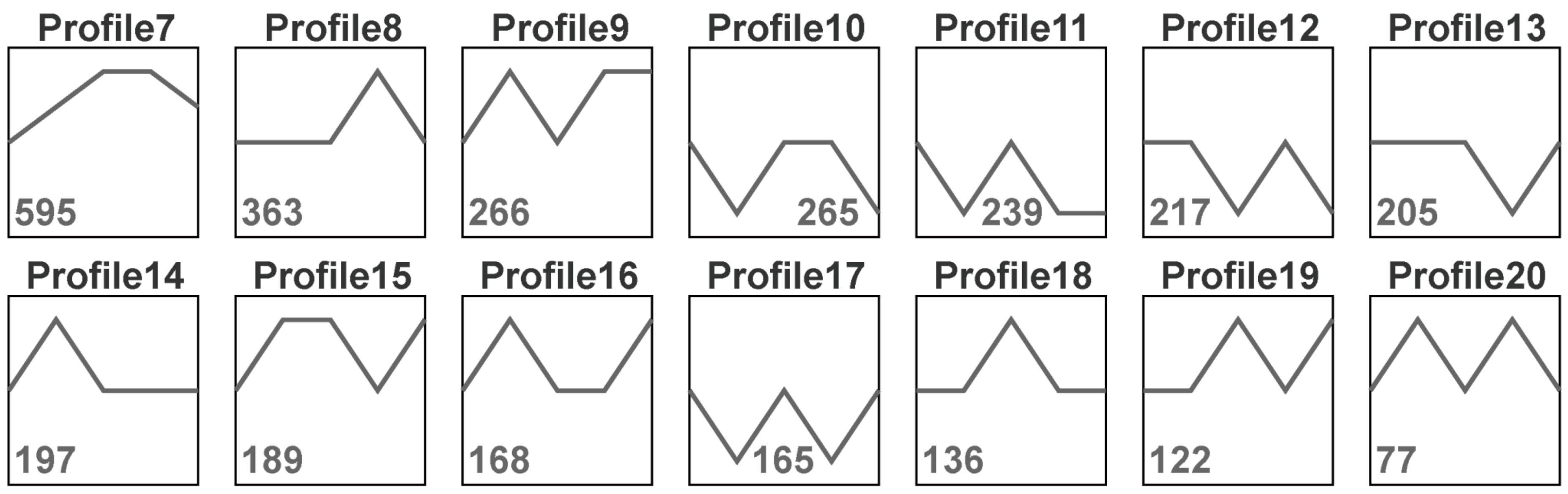

B

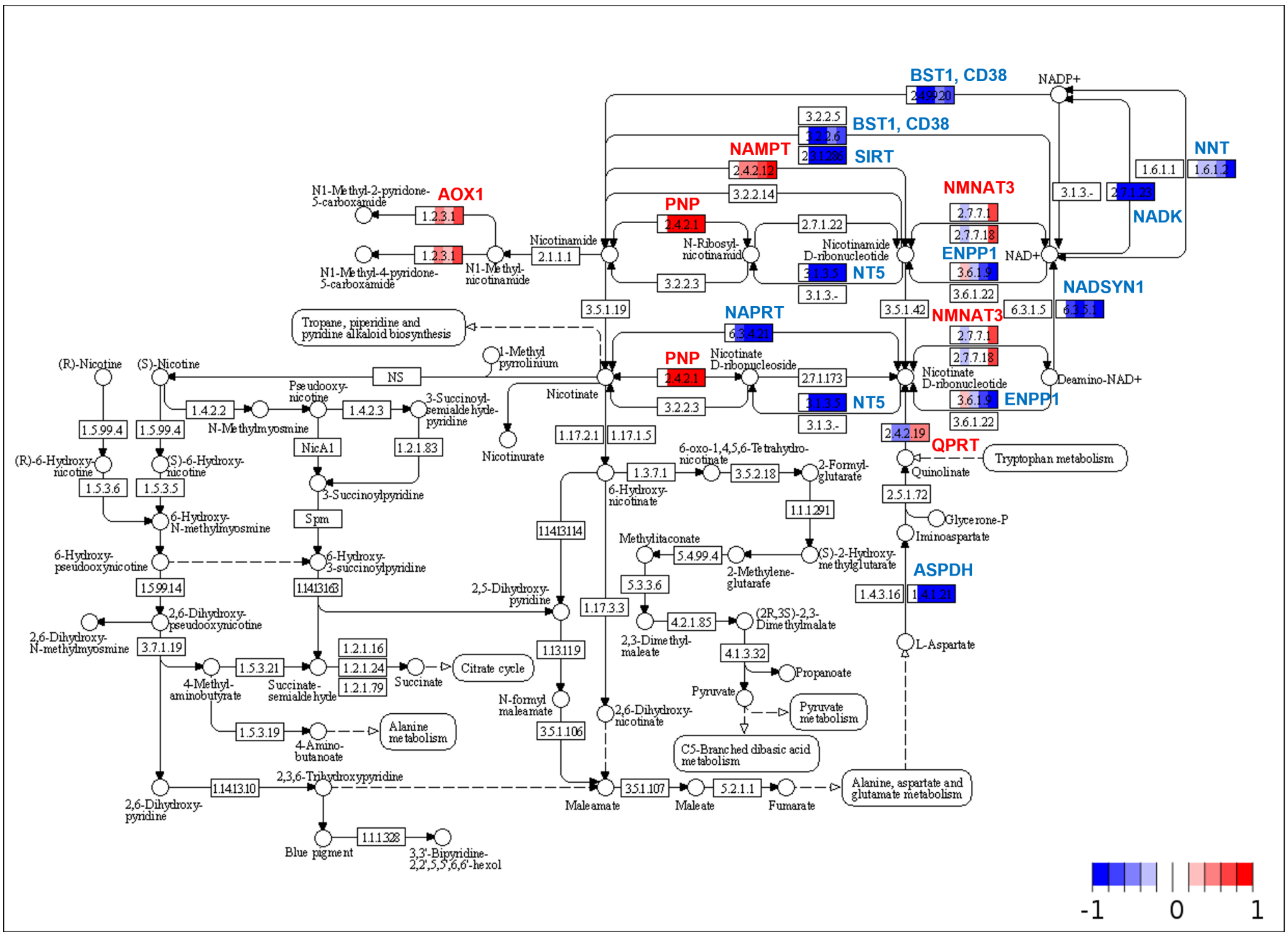

Figure S1 Differentially expressed gene analysis of KRAS^{G12D} overexpressed HPNE, related to Figure 1.

(A) Insignificantly enriched expression patterns of trend analysis of DEGs from Figure 1E. The polyline within each trend block represents the gene expression pattern, the number in the bottom left or right corner represents the number of genes included.

(B) Location and expression trends of differentially expressed genes in nicotinate and nicotinamide metabolic pathways. Each gene box was divided into 5 grids. From left to right, each grid represents the ratio of the gene expression level in HPNE KRAS/Off, HPNE KRAS/On 12 h, 1 d, 3 d, and 7 d cells, respectively, to the expression level in HPNE KRAS/Off.

Supplementary Figure S2

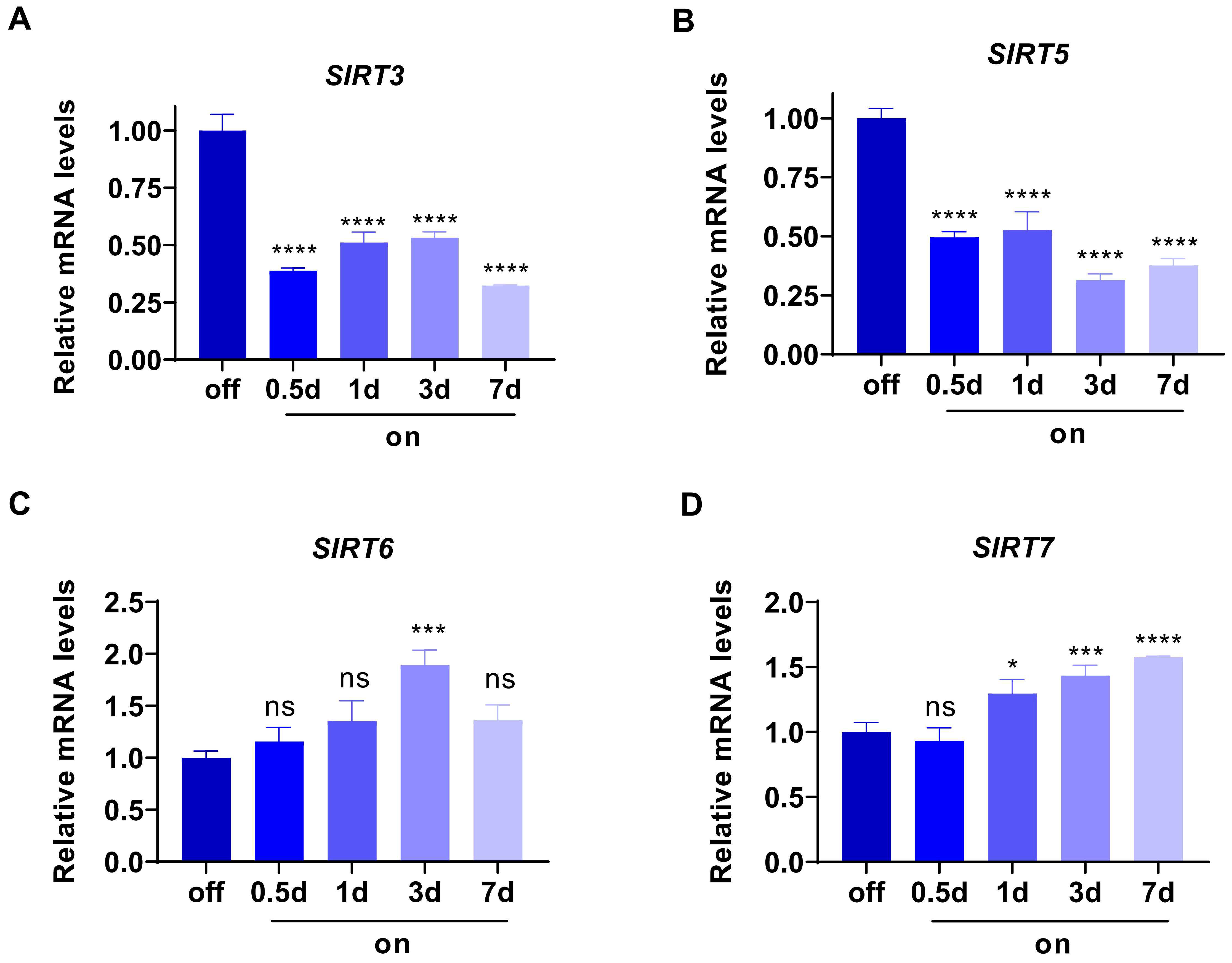

Figure S2. Effect of KRAS activation on the expression of Sirtuins, related to Figure 2. HPNE cells harboring inducible $KRAS^{G12D}$ vector were exposed to doxycycline to induce KRAS expression for various time points up to 7days as indicated, and mRNA expression levels of SIRT3 (A), SIRT5 (B), SIRT6 (C), and SIRT7 (D) were measured by qRT-PCR. Data were presented as relative levels (mean ± SD, n = 3) compared to the KRAS-Off cells. Student's t test was used to determine *p* values.. *, *p*<0.05; ***, *p*<0.001; ****, p<0.0001.

Supplementary Figure S3

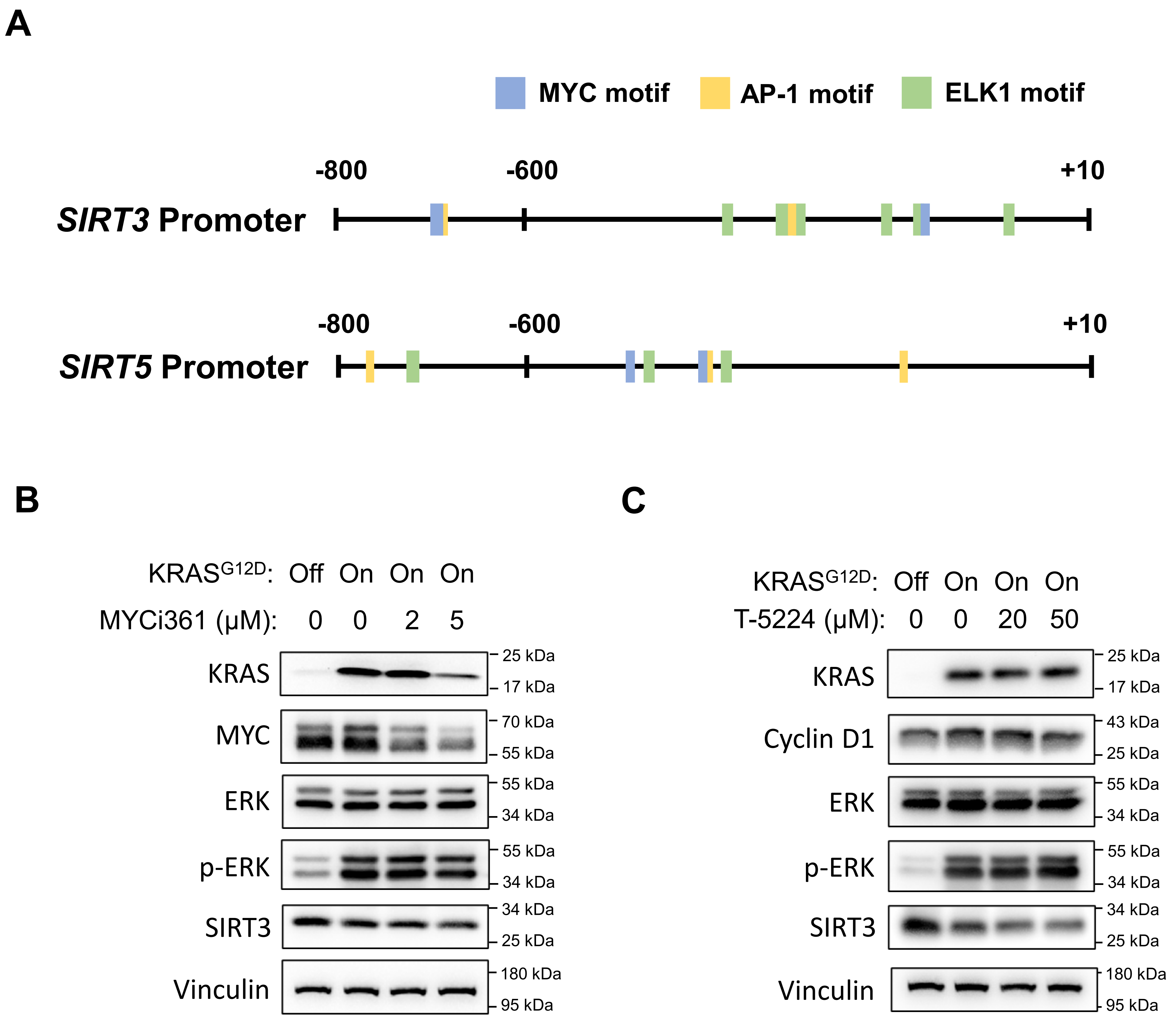

Figure S3. Investigation of potential regulators of SIRT3, related to Figure 2. (A) In silico analysis of KRAS-regulated transcription factor binding sites on SIRT3 and SIRT5 promoters. (B) KRAS, MYC, ERK1/2 and SIRT3 protein levels as well as the ERK1/2 phosphorylation levels in HPNE cells with KRAS/Off or KRAS/On treated with MYCi361 at indicated concentration for 24 h. Cell extracts were analyzed by Western blotting using vinculin as the loading control. (C)) KRAS, Cyclin D1, ERK1/2 and SIRT3 protein levels as well as the ERK1/2 phosphorylation levels in HPNE cells with KRAS/Off or KRAS/On treated with T-5224 at indicated concentration for 24 h. Cell extracts were analyzed by Western blotting using vinculin as the loading control.

Supplementary Figure S4

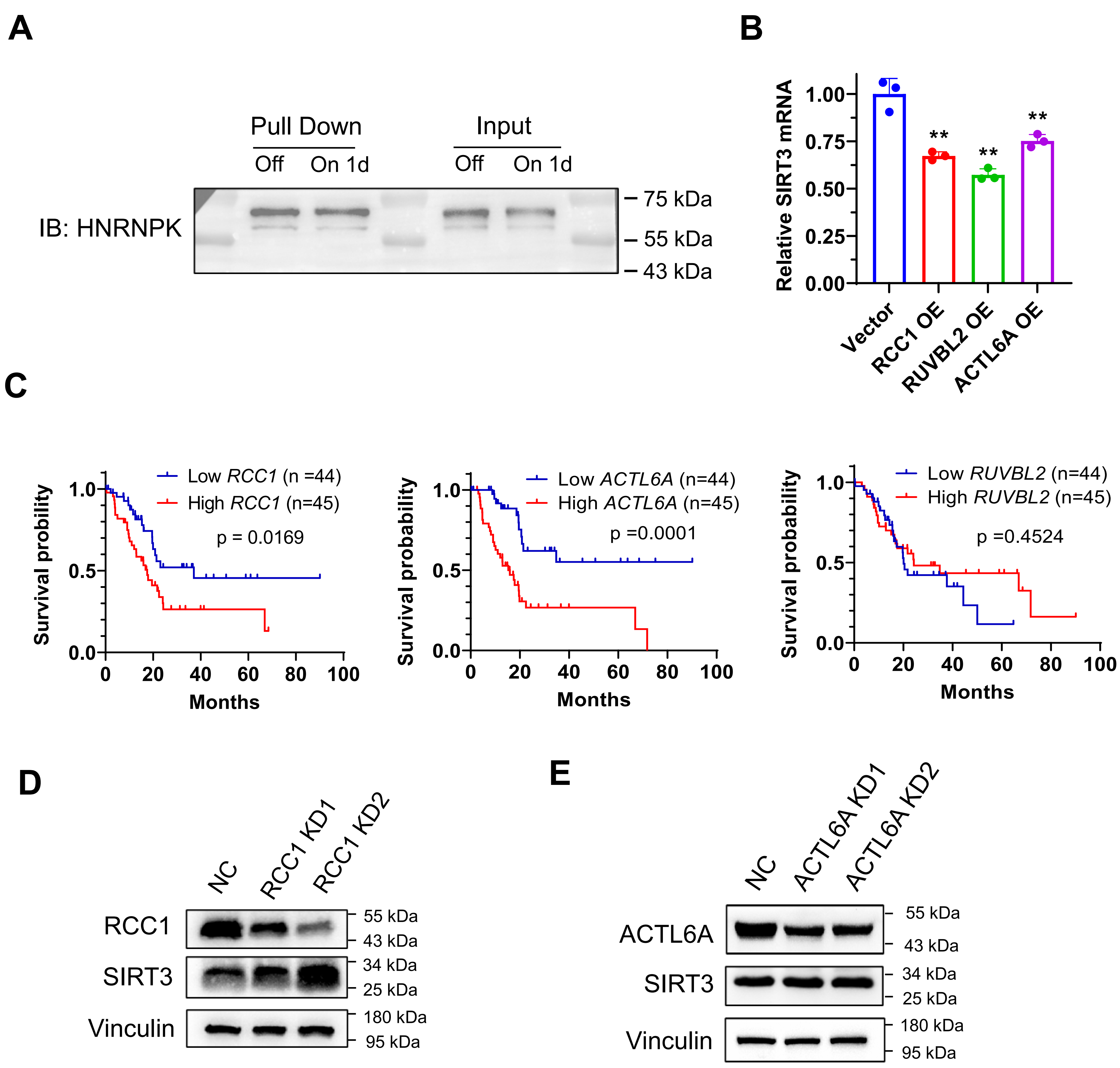

Figure S4. Identification of potential SIRT3 transcription regulators, related to Figure 3. (A) HNRNPK protein levels pulled-down by the SIRT3 promoter DNA probe from the nuclear protein extracts of the KRAS/Off or KRAS/On cells. (B) *SIRT3* mRNA levels in RCC1, RUVBL2 and ACTL6A overexpressed and control HPNE cells. The data are presented as mean ± SD (n = 3). p values were determined by Student's t test. **, p < 0.01. (C) Kaplan-Meier survival plot of pancreatic cancer patients with high and low *RCC1*, *RUVBL2* and *ACTL6A* expression at 75%/25% cutoff from the TCGA database. (D) Effect of RCC1 knockdown (KD) by siRNA on SIRT3 protein in HPNE cells. (E) Effect of ACTL6A knockdown (KD) by siRNA on SIRT3 protein expression in HPNE cells.

Supplementary Figure S5

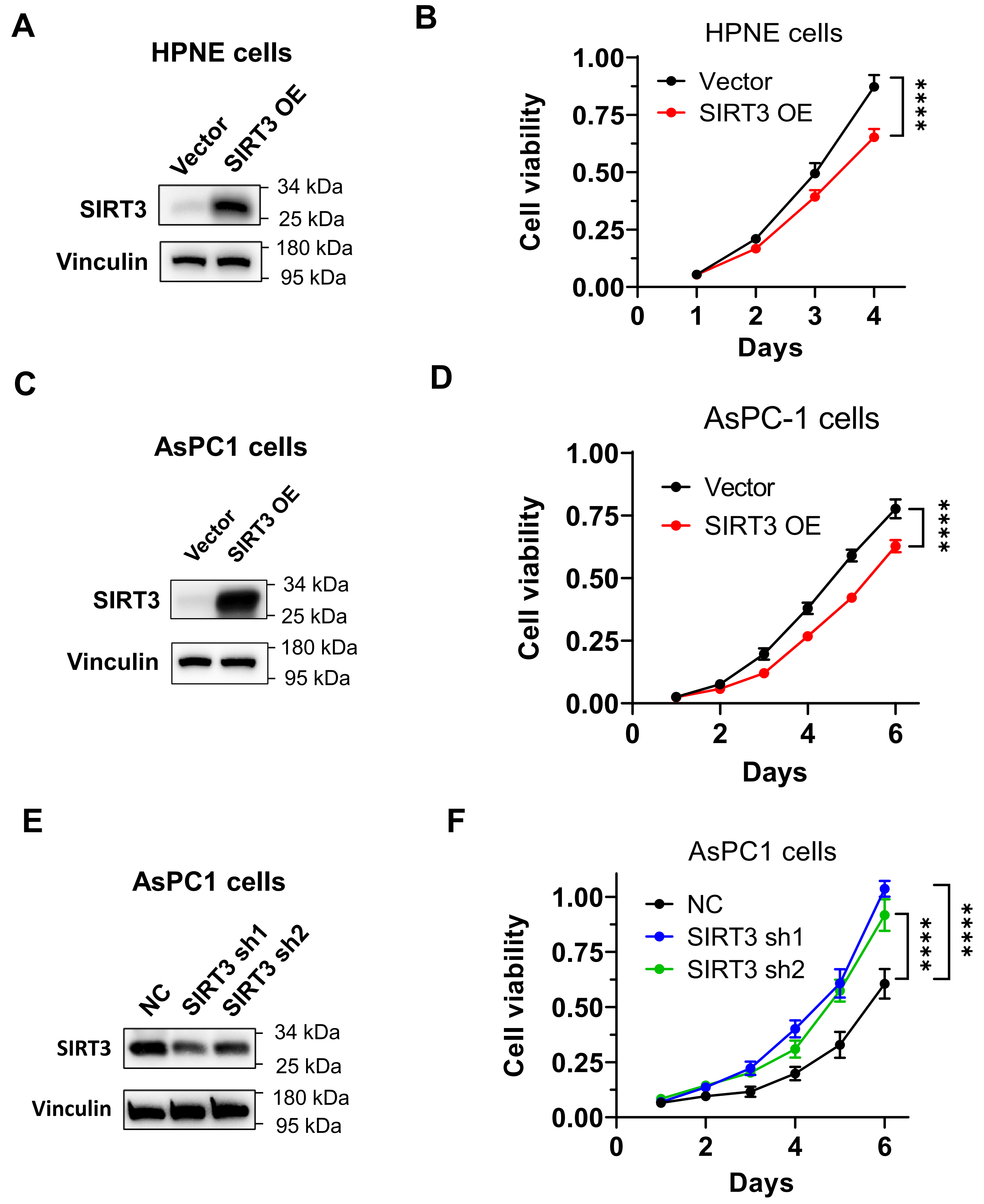

Figure S5. Effect of SIRT3 expression on cell proliferation, related to Figure 4. (A) SIRT3 protein levels in SIRT3 overexpressed (SIRT3 OE) and control (Vector) HPNE/On cells. (B) Growth curves of HPNE/On Vector cells and SIRT3 OE cells *in vitro*. The data are presented as mean ± SD (n = 3). *p* values were determined by Student's t test. ****, *p* < 0.0001. (C) SIRT3 protein levels in SIRT3 overexpressed (SIRT3 OE) and control (Vector) ASPC1 cells. (D) Growth curves of ASPC1 Vector cells and SIRT3 OE cells in vitro. The data are presented as mean ± SD (n = 3). *p* values were determined by Student's t test. ****, *p* < 0.0001. (E) SIRT3 protein levels in SIRT3 knockdown (SIRT3 sh1, sh2) and control (NC) AsPC1 cells. (F) Growth curves of AsPC1 NC, SIRT3 sh1 and sh2 cells *in vitro*. The data are presented as mean ± SD (n = 3). *p* values were determined by Student's t test. ****, *p* < 0.0001. In panels A and C, a short exposure time was used for the Western blotting due to the overexpression of SIRT3.

Supplementary Figure S6

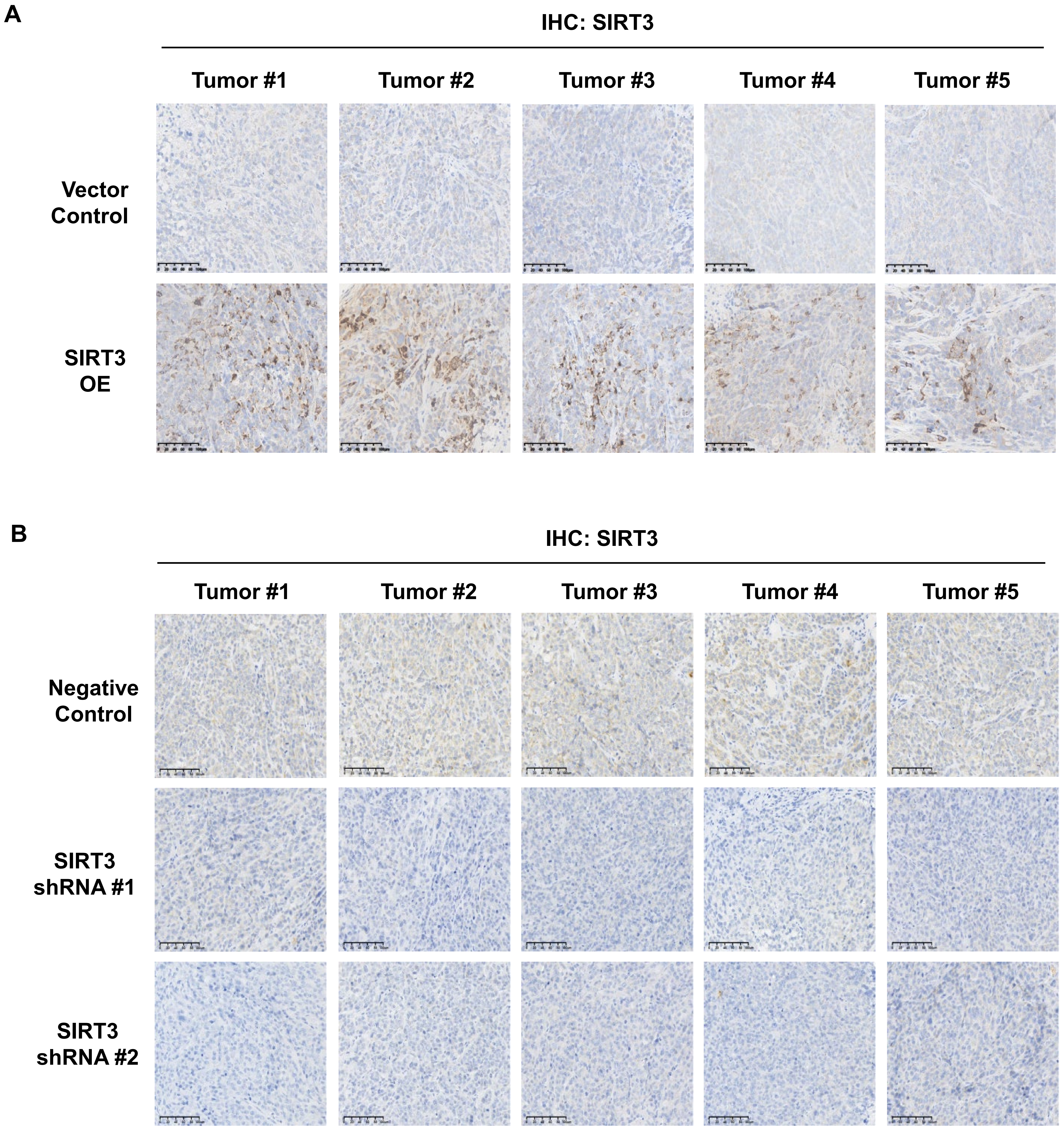

Figure S6. Immunohistochemistry (IHC) staining of SIRT3 in pancreatic tumor tissues, related to Figures 4. (A) SIRT3 IHC staining of tumor tissues from mouse xenografts inoculated with PANC1 cells transfected with control vector or SIRT3 overexpression (OE) vector as indicated. The scale bar represents 100 μm. (B) SIRT3 IHC staining of tumor tissues from mouse xenografts inoculated with PANC1 cells transfected with negative control shRNA or SIRT3 shRNA (#1 or #2) as indicated. The scale bar represents 100 μm.

Supplementary Figure S7

A

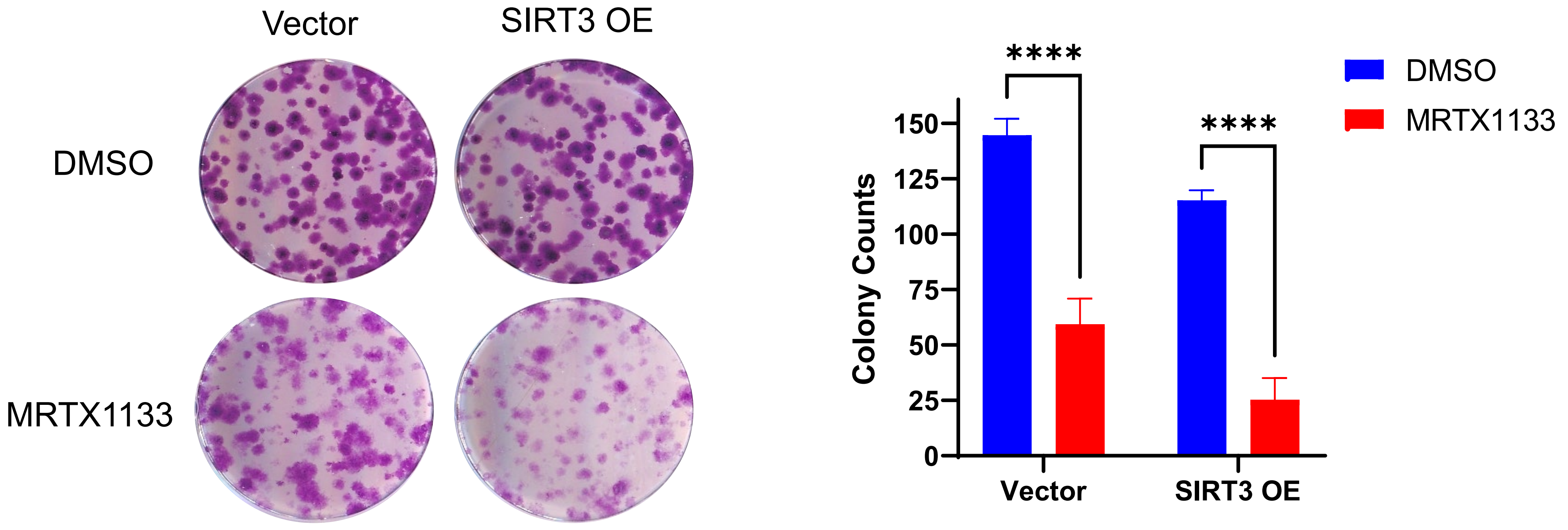

Group	Inhibition by MRTX1133 (%)	p
Vector	59.0±8.0	
SIRT3 OE	78.0±8.4	0.047

B

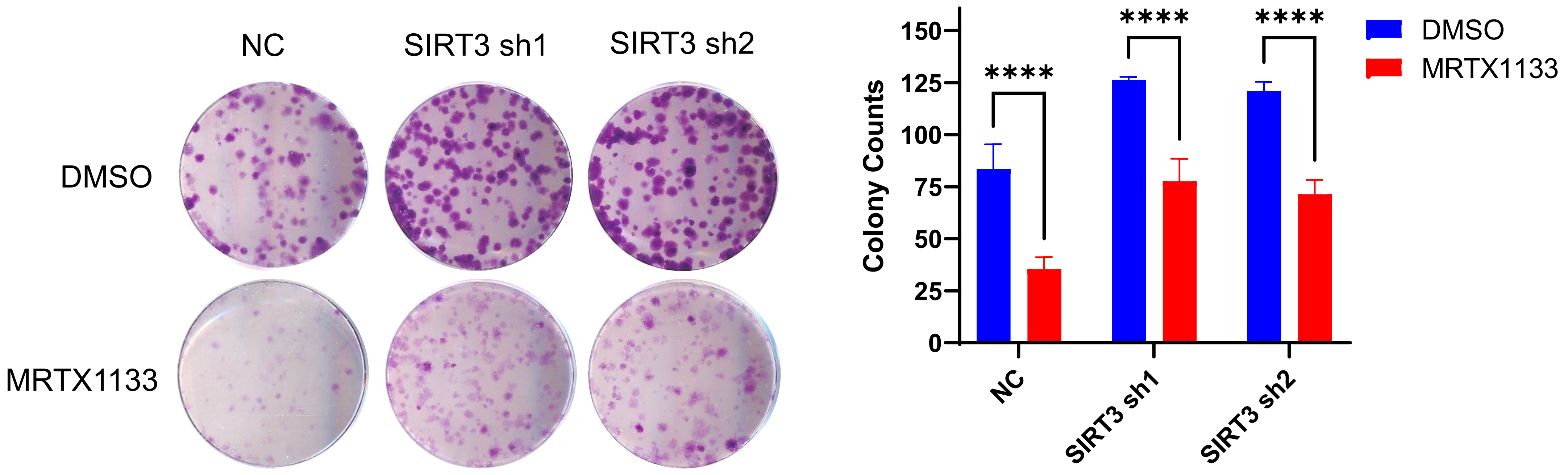

Group	Inhibition by MRTX1133 (%)	p
NC	57.8±7.0	
SIRT3 sh1	38.5±8.5	0.039
SIRT3 sh2	41.0±5.8	0.033

Figure S7. Effect of SIRT3 expression in pancreatic cancer cells on their sensitivity to KRAS^{G12D} inhibitor, related to Figure 4. (A) PANC1 cells transfected with SIRT3 overexpression (OE) or with the control vector were treated with the KRAS^{G12D} inhibitor MRTX1133 (1 μM). Cytotoxicity was measured by colony formation assay for 14 days. Representative images of cellular colonies were shown on the upper left panel; colony counts were shown on the upper right panel; Different sensitivity between the control and SIRT3-OE cells was compared using colony counting. (B). PANC1 cells transfected with SIRT3 shRNA (SIRT1 sh1, SIRT3 sh2) or with control shRNA (NC) were treated with the KRAS^{G12D} inhibitor MRTX1133 (1 μM). Cytotoxicity was measured by colony formation assay for 14 days. Representative images of cellular colonies were shown on the upper left panel; colony counts were shown on the upper right panel; Different sensitivity between the control and SIRT3-sh KD cells were compared using colony counting. Data are presented as mean ± SD (n = 3). p values were determined by Student's t test. *, $p < 0.05$; **, $p < 0.01$.

Supplementary Figure S8

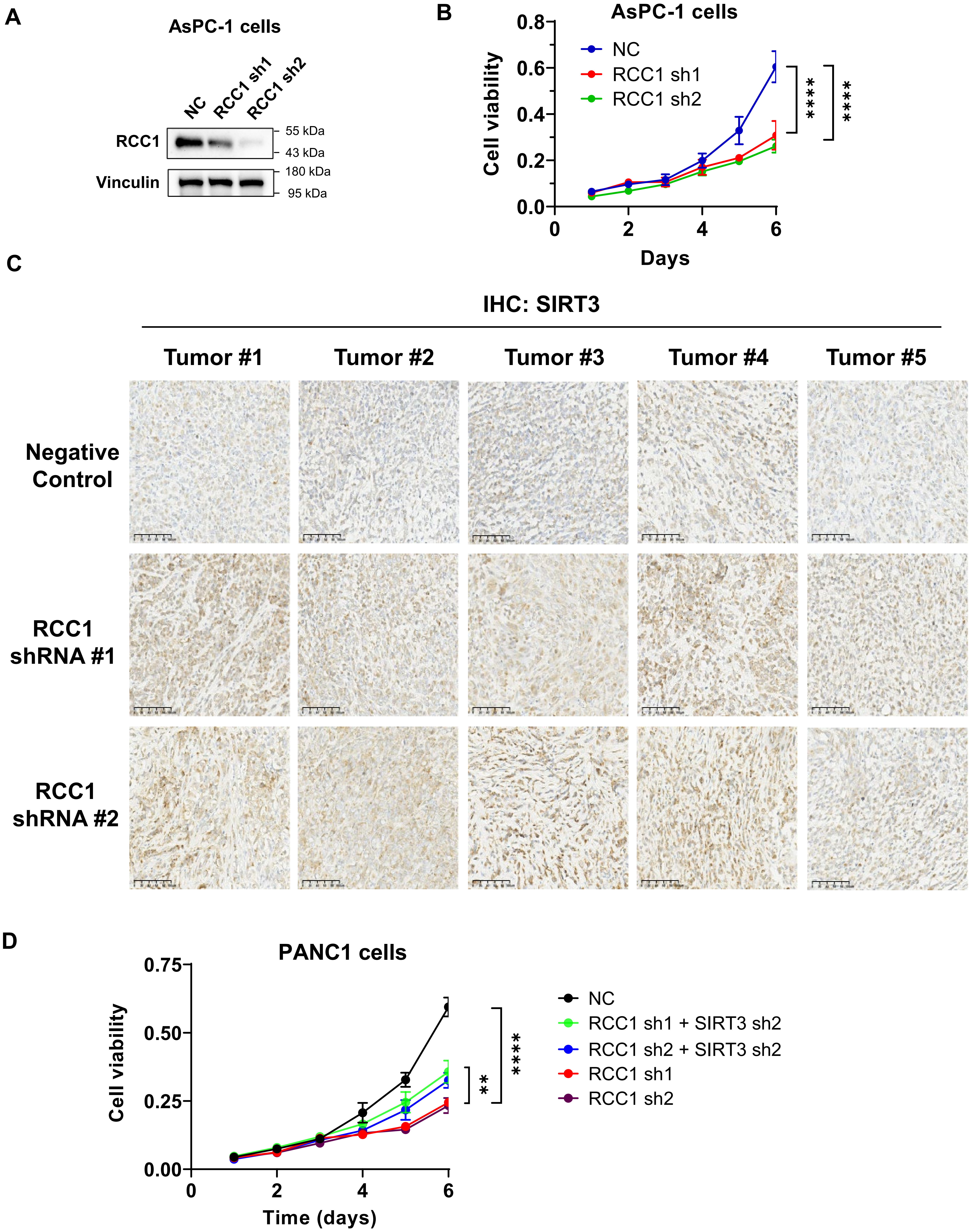

Figure S8. Immunohistochemistry (IHC) staining of SIRT3 in pancreatic tumor tissues with RCC1 knockdown, related to Figures 5. (A) SIRT3 protein levels in RCC1 knockdown (RCC1 sh1, sh2) and control (NC) AsPC1 cells. (B) Growth curves of AsPC1 NC, RCC1 sh1 and sh2 cells *in vitro*. The data are presented as mean ± SD (n = 3). *p* values were determined by Student's t test. ****, $p < 0.0001$. (C) SIRT3 Immunohistochemistry staining of tumor tissues from mouse xenografts inoculated with PANC1 cells transfected with negative control shRNA or RCC1 shRNA (#1 or #2) as indicated. The scale bar represents 100 μm. (D) Growth curves of PANC1 NC, RCC1 sh1, RCC1 sh2, RCC1 sh1 + SIRT3 sh2 and RCC1 sh2 + SIRT3 sh2 cells *in vitro*. The data are presented as mean ± SD (n = 3). p values were determined by Student's t test. **, $p<0.01$; ****, $p < 0.0001$.

Table S1 siRNA and shRNA sequences for specific gene knockdown

siRNA/shRNA	Target sequence
SIRT3 sh1	CCCAACGTCACTCACTACTTT
SIRT3 sh2	GTGGGTGCTTCAAGTGTTGTT
RCC1 sh1	CCAGAACCTAACATCCTTCAA
RCC1 sh2	CCTGGCTTTGCCTACTAGAAA
shNC (Negative control)	CCTAAGGTTAAGTCGCCCTCG
RCC1 siRNA1	GUCUAUUCCUUCGGCUGCAAUTT
RCC1 siRNA2	CUGCAUGGAUUCGGAAGGAAATT
ACTL6A siRNA1	CACCUACUACAUAGAUACUAATT
ACTL6A siRNA2	CGGUACUUCAAGUGUCAGAUUTT
NC siRNA (Negative control)	UUCUCCGAACGUGUCACGUTT

Table S2 Primer sequences for qRT-PCR

Primer	Sequence (5' to 3')
SIRT3 Forward	GACATTCGGGCTGACGTGAT
SIRT3 Reverse	ACCACATGCAGCAAGAACCTC
KRAS Forward	GAGTACAGTGCAATGAGGGAC
KRAS Reverse	CCTGAGCCTGTTTTGTGTCTAC
RCC1 Forward	AAAGCAAGAAGGTGAAGGTCTCA
RCC1 Reverse	CATCACATTCTCACCCAGCCC
RUVBL2 Forward	GACTACGACGCTATGGGCTC
RUVBL2 Reverse	TGGGTGCGAGAGTTGATGAC
ACTL6A Forward	GGGGAGATGAAGTTGGAGCC
ACTL6A Reverse	AGGAAAATCCACCTTGGGGC
ACTB Forward	CATGTACGTTGCTATCCAGGC
ACTB Reverse	CTCCTTAATGTCACGCACGAT
Forward Primer for ChIP assay	GGAAGCAGTGAATCTCAGACCG
Reverse Primer for ChIP assay	GAGGAACCTTGAGTGGCAGG
